# Aberrant neuronal firing: a paracrine route to glioblastoma expansion

**DOI:** 10.1186/s12964-025-02404-8

**Published:** 2025-09-25

**Authors:** Ji Yeon Lee, Bon Il Koo, Trang Huyen Le-Kim, Yoonsung Nam

**Affiliations:** 1 Department of Biological Sciences, Korea Advanced Institute of Science and Technology, Daejeon, Republic of Korea; 2 Department of Materials Science and Engineering, Korea Advanced Institute of Science and Technology, Daejeon, Republic of Korea

**Keywords:** Glioblastoma, Neuron-Tumor crosstalk, Coculture platform, Electrical stimulation, Neuronal excitation, Hyperexcitation, Neurotransmitter, Recurrence

## Abstract

**Background:**

Glioblastoma multiforme (GBM) is a highly aggressive astrocytic glioma with a devastating survival rate of less than 7%. Despite treatment with surgical resection and chemoradiotherapy, a majority of GBM cases recur. The intricate tumor microenvironment and the elusive nature of its recurrence are still controversial. Herein, we explore the role of neuronal hyperstimulation in glioblastoma cell regrowth post-chemotherapy, focusing on cancer-neuron interactions.

**Methods:**

A direct electrical stimulation system, validated by COMSOL Multiphysics simulation, was used to induce stimulation of neuronal networks through the formation of an extremely low frequency (ELF) electric field, and changes by excitability were tracked. The custom-designed co-culture system, enabling the sharing of paracrine signals in an independent microenvironment cultivation of neuronal networks and glioblastoma cell, was employed to investigate the effects of neuronal excitability on glioblastoma cell.

**Results:**

Power-frequency electric fields are applied to hippocampal neuronal networks to elicit abnormal neuronal activity, evidenced by calcium influx and neurotransmitter release. While temozolomide effectively suppresses glioblastoma cell proliferation, their co-culture with stimulated neurons reignites cancer growth. Blocking glutamate release from neuron networks counter the effects of neuronal activity, highlighting the significance of paracrine signaling in glioblastoma cell proliferation and recurrence.

**Conclusions:**

Our findings illuminate a pathway through which environmental factors contribute to GBM regrowth following chemotherapy and propose a potential therapeutic target, neuron-cancer communication, to prevent GBM recurrence.

**Supplementary Information:**

The online version contains supplementary material available at 10.1186/s12964-025-02404-8.

## Introduction

Glioblastoma multiforme (GBM) remains a major challenge in oncology, characterized by aggressive behavior and poor prognosis. Despite recent advances in treatment techniques, the survival rates for GBM patients remain abysmal. GBM is notoriously resistant to standard-of-care treatments, including surgery, radiotherapy, and chemotherapy, and its recurrence is nearly universal - about 90% of patients experience tumor regrowth within two years of initial diagnosis [1–3]. The GBM’s persistence can be attributed to both its substantial inter- and intra-tumoral heterogeneity and the complex interplay within the tumor microenvironment (TME), particularly the intricate crosstalk between cancer cells and neuronal components [4–6]. Unveiling this crosstalk is not just an academic interest but constitutes an urgent clinical need, given its potential implications to mitigate tumor recurrence and overcome resistance to therapy.

In recent works, the complex bidirectional communication between neurons and cancer cells has been highlighted, suggesting a possible influence of neuronal activity on tumor growth and survival. In particular, neuronal activity is suspected as a hitherto underappreciated yet significant contributor to GBM progression. The activation of excitatory synapses between adjacent neurons can promote tumor colonization, migration, and proliferation in gliomas by intercepting neuronal communication signals. Venkatesh et al. observed synaptic gene expression and the formation of structural synapses that are dependent on the neuronal action potential in a subset of human glioma cells transplanted into mouse brains, suggesting similarity to the established mechanisms of neuronal synapses [7]. Venkataramani et al. identified a distinct structure of neuronal excitatory synapse in gliomas, with adjacent presynaptic neurons containing a reservoir of neurotransmitter vesicles at the presynaptic membrane domain, as revealed by electron microscopy imaging in a human glioma xenograft mouse model [8]. Furthermore, Zeng et al. correlated tumor progression with signal transmission by comparing the survival of mice injected with breast cancer cells engineered to downregulate glutamate receptor expression against those injected with unmodified breast cancer cells [9]. Various theories regarding the precise molecular mechanisms orchestrating this interaction, have been proposed, but a definitive understanding especially within the CNS, remains elusive. This knowledge gap represents a critical barrier to devising new therapeutic strategies aimed at modulating the TME in the treatment of GBM.

Previous studies used optogenetic and chemical stimulation methods as neural stimulation sources, but these are very few stimulation factors that are difficult to encounter in real life [8, 10]. On the other hand, electrical stimulation, especially the extremely low frequency (ELF), can be easily accessed not only in real life by electricity systems commonly in the power-frequency range (50–60 Hz), but also through devices for patients’ medical activities [11]. Biologically vulnerable individuals may be more susceptible to externally applied ELF electric fields, particularly those that influence or modulate central nervous system activity, and it appears to be a neuropathologically suitable stimulus for our research exploring the crosstalk of cancer-neuron. Additionally, the World Health Organization (WHO) continues to explore the health effects of exposure to the ELF electromagnetic field, but still no conclusion by only limited evidence restricted to a single study or present unresolved questions.

Our work explores the possibility that exposing neurons to ELF electric fields may influence the growth and survival mechanisms of GBM cells via paracrine signaling. The effects of ELF exposure, especially at levels present in our environment, on the biological behaviors of brain cancer cells remain unexplored. Specifically, the interaction between neurons and glioma cells within the TME under such conditions has not been examined. In this work, by combining an in vitro co-culture system with an electrochemical setup, we simulated the neuronal-GBM interactions and dissected the consequences of induced neuronal excitability on GBM cell dynamics. We aimed to clarify the effects of paracrine signaling triggered by ELF electric stimulation, especially regarding the secretion of neurotransmitters such as glutamate, and their influence on cancer cell growth. This work can shed light on the potential of neuronal activity as a modulator of GBM progression and treatment resistance. Furthermore, our investigation not only contributes to our understanding of the environmental impact on GBM biology but also contributes to a new paradigm of treatment by proposing new strategies for therapeutic intervention, with the potential to impede tumor progression and recurrence by attenuating the malignant potential of the TME.

## Results

### Co-culture system designed to mimic glioblastoma microenvironment under hyperexcited neuronal networks

**Fig. 1:**
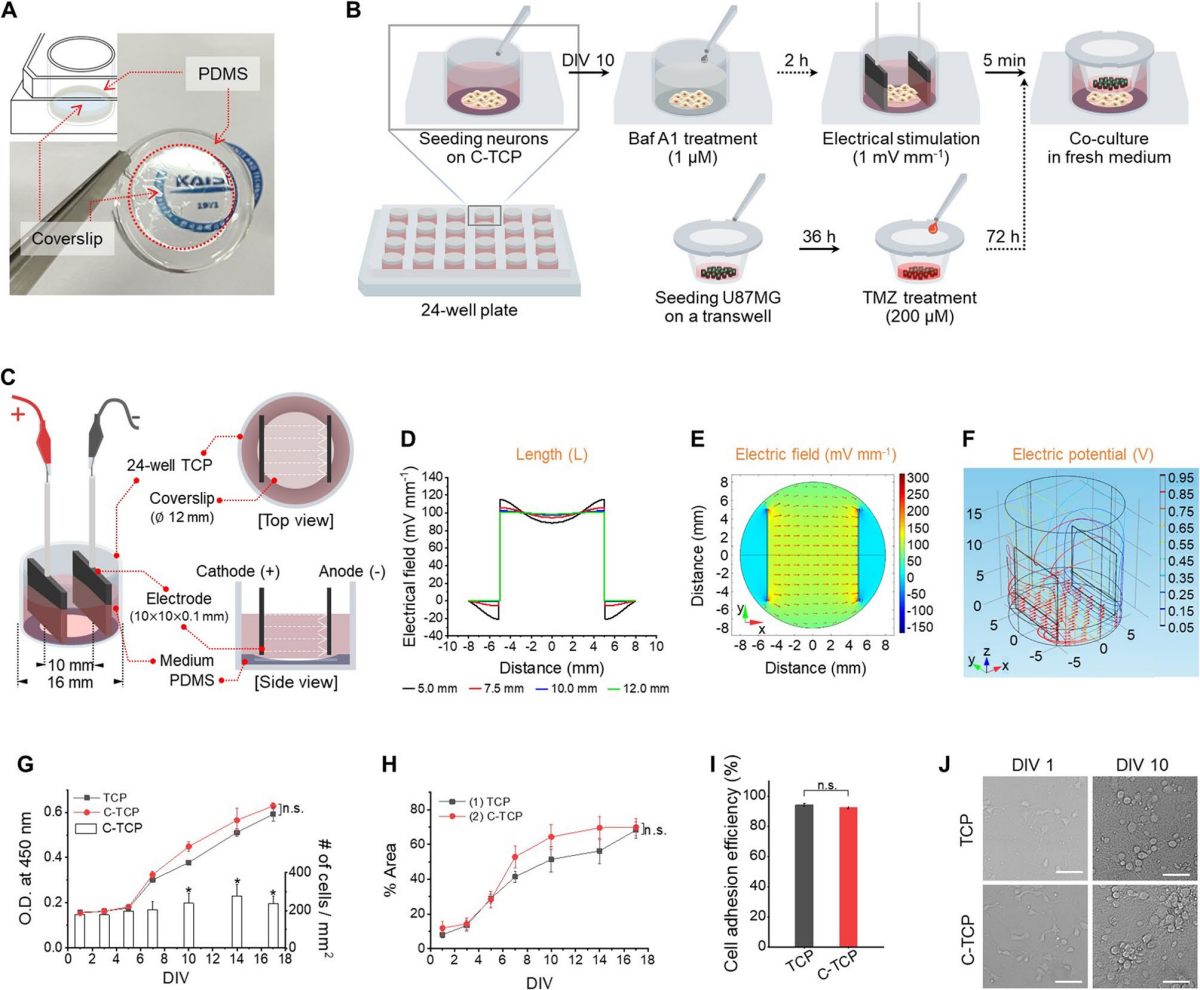
Schematics of the neuron-to-glioblastoma interaction study setup. **A** Schematic overview showing the experimental design for co-culture. **B** Photograph and schematic illustration of the coverslip-attached tissue culture plate (C-TCP) for neuronal cell culture. The part indicated by the dotted line is performed selectively according to the experimental group. **C** Schematic description of electrical stimulation setup using custom C-TCP with details of the apparatus. **D-F** A plot of the electric field distribution with different lengths of Pt-electrodes (**D**), the 2D electric field distribution (**E**) and 3D electric potential (**F**) were performed using the AC/DC mode of COMSOL Multiphysics software for finite element analysis. The range of electric field (mV mm^−1^) and electric potential (V) are shown with the corresponding color bars in each panel. **G** Quantitative results of CCK-8 and number of cells in primary neuronal cells. Data were presented as mean ± standard deviation (SD). One-way ANOVA with Tukey’s post hoc test for comparisons. ^*^p < 0.05 indicates statistical significance compared with DIV 1. n.s., not significant (*p* > 0.05). **H** Percentage of the cell coverage area determined from microscopic images. Data were presented as mean ± standard deviation (SD). n.s. = not significant (*p* > 0.05) by one-way ANOVA with Tukey’s post hoc test for comparisons. **I** Cell adhesion efficiency at 24 h after seeding. Data were presented as mean ± standard deviation (SD). n.s. = not significant (*p* > 0.05) by one-way ANOVA with Tukey’s post hoc test for comparisons. **J** Microscopy images of primary neuron cells for demonstrating arborization/proliferation by tracking morphological changes. Scale bars: 50 μm

A detachable substrate for neuronal network culture was constructed by placing a coverslip on PDMS post-placed in individual wells of a 24-well tissue culture plate (TCP) (Fig. 1A). The coverslip attached TCP (C-TCP) design, enabled the detachable feature, allows us to retrieve the substrate for immunocytochemical staining and cell imaging, which was previously unable to perform with conventional TCP. Figure 1B introduced a transwell-based co-culture system employed to examine the effects of paracrine signals from neuronal networks on glioblastoma cells. The co-culture procedures were adjusted to incorporate inhibitors, chemotherapy treatments, and electrical stimulation. An electrical stimulation apparatus equipped with two electrodes of platinum, known to produce negligible cytotoxic byproducts, was used to uniformly excite neuronal networks (Fig. 1C) [12].

Computational simulations of electrode dimensions in different lengths and widths were performed to determine optimal configurations (Fig. 1D; Figure S1A-C, Supporting Information). The simulation environment was modeled to assess the effects at an applied voltage of 1 V. The electrode lengths of 10 and 12 mm confirmed a uniform electric field distribution across the culture plate surface, whereas lengths below threshold induced electric field fluctuations. Electrode thickness, however, did not markedly influence the electric field characteristics. The electrode with 10.0 mm×0.10 mm was selected as it produced a stable electric field intensity of 100 mV mm^−1^ (Fig. 1E), sufficient to affect neurons as previously reported [13, 14]. Three-dimensional simulations also showed a uniform potential distribution within the medium, characterized by the magnitude (strength of the electric field) and orientation (directional flow) of the vectors (Fig. 1F). In addition, a uniform decrease in the electric potential across the medium was quantitatively presented in Figure S1D-E (Supporting Information). Taken together, the resulting directional electric potentials from our design are thought to be suitable for neuronal cell stimulation, suggesting their applicability in neurobiological studies.

Next, we assessed the biocompatibility of C-TCP to examine any potential side effects from the substrates that could interfere with our interpretations of neuron-cancer interactions. Multiple parameters were evaluated using both of the cell types used in this work: human glioblastoma cell line and primary neurons derived from late-stage murine embryonic hippocampal tissues (Figure S2A, Supporting Information). Hippocampal neurons are considered a robust model for neurobiological research due to large pyramidal neuron populations and relative ease of isolation with minimal glial cell contamination [15, 16].

Results from the CCK-8 and neuron cell counts are presented in Fig. 1G and Figure S2B-G (Supporting Information). Despite seeing a significant increase in cell number after days-in-vitro (DIV) 10, the absorbance for neurons cultured on C-TCP and TCP constantly increased after DIV 5, consistent with the establishment of neuronal networks and the extension of neurites and dendrites, rather than cellular proliferation. This expansion was corroborated by a rapid increase in the cell coverage area, signifying the formation of multiple dendrites starting from DIV 5 by progress from axon formation to dendrite formation on the maturation process, as shown in Fig. 1H [17]. Neuronal cell adhesion efficiency on C-TCP was 92.28 ± 0.7%, with minimal cell death, comparable to that on TCP (94.14 ± 0.96%) (Fig. 1I-J). Moreover, the neurons clustering and network formation were evident from DIV 10 to 17 (Figure S3A, Supporting Information). Consequently, DIV 10 was identified as the point at which neuronal networks on C-TCP reached maturity, establishing it as the appropriate stage for initiating co-culture in subsequent electrical stimulation studies without inducing cytotoxicity [18–20]. The stable astrocyte-to-neuron ratio in DIV 10 was confirmed by immunostaining by using antibodies for specific markers of MAP2 (microtubule-associated protein 2) and GFAP (glial fibrillary acidic protein), antibodies revealed neurons and astrocyte cells, respectively (Figure S3B, Supporting Information) [21]. It is at a comprehensible level comparable to the findings of Sahu et al. in their study on the entire cell population in primary hippocampal neurons [22]. In addition, it can be inferred that the increase in cell number at DIV 10 was due to the influence of astrocytes.

Unlike neurons, which exhibit increased arborization over time in culture, U87MG exhibited fast proliferation on both TCP and C-TCP, with no significant difference in growth rates (Figure S3C-D, Supporting Information). Cell adhesion efficiency was assessed 24 h post-seeding, prior to the U87MG doubling time of 36 h, to prevent confounding effects from cell division [23]. Adhesion rates were high for both TCP and C-TCP, with no statistically significant difference, indicating that both substrates support favorable initial cell attachment, essential for subsequent cell proliferation (Figure S3E, Supporting Information). Additionally, no morphological differences were observed between cells on TCP and C-TCP (Figure S3F, Supporting Information), suggesting that C-TCP is a suitable substrate for developing neuronal networks and for co-culture studies with U87MG.

We conducted monocultures of neurons and U87MG in the media outlined in Table S1. We used an elimination approach to determine their suitability for studying physiological neurotransmitter release/uptake and the impact of neurotransmitters and neurotrophic factors (Figure S4, Supporting Information). Consequently, B-2 was selected for further experiments due to its capacity to delay U87MG growth while preserving neuronal and U87MG morphology.

### Electrical stimulation modulates physiological signal of neuronal networks

Subsequent experiments focused on neuronal network activities by examining Ca^2+^ uptake to electrical stimuli in terms of voltage, duration, and frequency. The neuronal responses follow a sequence initiated by an action potential that causes Na^+^ channels to open, leading to depolarization of the presynaptic cell, followed by the opening of Ca^2+^ channels. Subsequent Ca^2+^ uptake triggers a signaling cascade that promotes vesicle formation and the exocytosis of neurotransmitters [24, 25]. Therefore, Ca^2+^ uptake has been widely used to assess spatiotemporal electrophysiological dynamics [26, 27]. Abnormal high-frequency burst firing patterns in neuronal networks were identified, and their relevance to neuropathology has been reported [28–32]. Qualitative assessments included analyses of representative Ca^2+^ images, Ca^2+^ wave responses (F/F_0_), sequential nuclear firing patterns, and shifts in calcium within neuronal networks [33]. For quantitative analysis of spontaneous Ca^2+^ activity, forty regions of interest (ROI) were selected in the neuronal soma from representative images. Quantitative analyses encompassed the number of firing peaks per cell, onset timing, signal periodicity, signal duration, the full width at half-maximum (FWHM), and relative peak amplitude in terms of calcium fluorescence [34, 35].

In the group without stimulation (control) occurred the distinct Ca^2+^ signals, albeit weak, that indicated mature neuronal networks capable of signal propagation [36]. Frequency control for physiological characteristic simulation based on 100 mV mm^−1^ for 5 min, which is the minimum effective stimulation condition for neural network activation without physiological damage, induced calcium transients and nuclear firing under conditions below 100 Hz, and a lower proportion of unresponsive cells was observed compared to the control (Figure S5-8, Supporting Information). These results suggest that frequencies above 5 Hz markedly increase cellular activity, consistent with the theory that synchronized depolarization in neuronal networks triggers firing [37, 38].

**Fig. 2:**
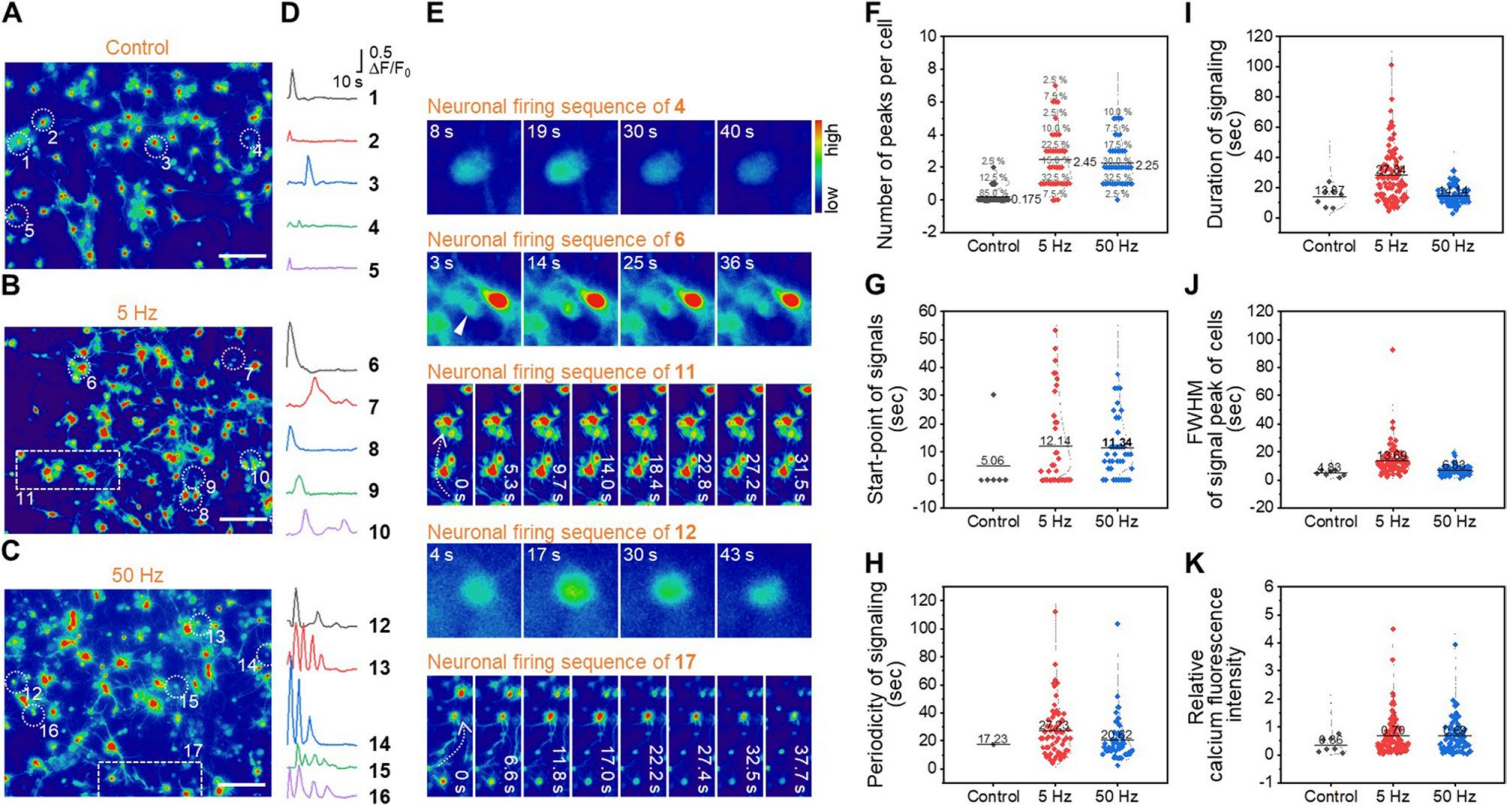
Calcium ion imaging of electrical stimulation mediated neuronal network firing. **A-C** Representative calcium ion images of five randomly selected tracing cells without (control) (**A**) and with electrical stimulation under 5 (**B**) and 50 Hz (**C**). Scale bars: 100 μm. **D** Fluorescence responses (∆F/F_0_) taken from the selected region of interest (ROI) in **A-C**. **E** Fluorescence micrographs of calcium ion fluctuations (square images) and intracellular calcium ion shifting in cell network interaction (rectangular images) at specific positions as numbered above the image. The color bar indicates the scale of relative fluorescence intensity. A white arrowhead indicated the cells with a neuron firing signal. The dotted arrows in the image are the direction of calcium ion shifting. **F-K** Calcium ion signal analysis without and with electrical stimulation (5 and 50 Hz): the number of firing peaks per cell (**F**), starting time point of the signal (**G**), periodicity of cell signaling (**H**), duration of cell signaling (**I**), the full width at half-maximum of firing peak of the cell (**J**), and relative calcium fluorescence intensity (**K**). The black bar is the mean value (*n* = 40)

At 50 Hz, poly-spiked neuronal activity potentially associated with abnormal physiological behaviors was observed (Fig. 2A-D). To discern the effects of stimulation on neuronal networks further, 5 Hz electrical stimulation was applied, which resulted in mono-spiked signals and avoided pathological responses. Both 5 and 50 Hz stimulation demonstrated calcium dynamics corresponding to chained neuronal depolarization, as seen in sequential imaging, with activation confirmed by the number of peaks per cell and signal onset timing (Fig. 2E-G). Higher signal onset points from 1 to 50 Hz suggested true neuronal activation, rather than laser-induced artifacts. The characteristic poly-spike features, including the narrow FWHM, duration, and signal periodicity, were recorded (Fig. 2H-J), along with sharp waveforms and shorter intervals between bursts, implying rapid depolarization and signal propagation (Figure S8A-B, Supporting Information). Calcium fluorescence intensity was substantially higher at 5 and 50 Hz compared to unstimulated conditions (Fig. 2K), indicating a robust neuronal response. Overall, our findings suggest that frequency-modulated electrical stimulation leads to significant electrophysiological changes at the cellular level and synchronous network activity, with 50 Hz specifically evoking neuropathological responses.

### Glutamate exocytosis is associated with level of neuronal activation

**Fig. 3:**
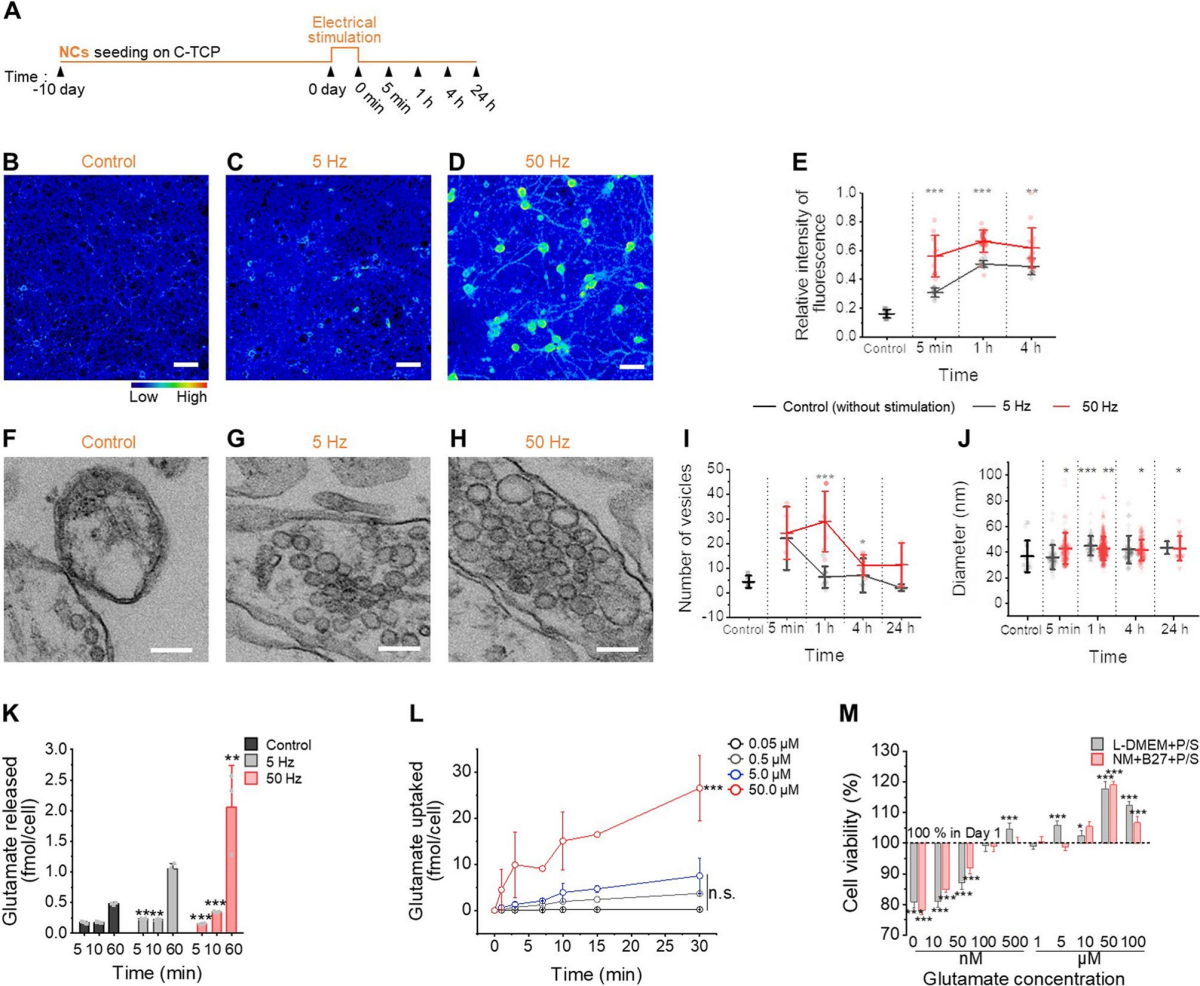
Electrical stimulation-induced neuronal network firing responses in a glutamate-deplete environment. **A** Diagram of the experimental design for analysis of firing neuronal network under glutamate-deplete environment. **B-D** Fluorescence micrographs of glutamate in the neuronal network without electrical stimulation (control) (**B**) and after 5 min from electrical stimulation under 5 Hz (**C**) and 50 Hz (**D**). The color bar indicates the scale of relative fluorescence intensity. Scale bars: 50 μm. **E** Quantified graph showing the relative intensity of intracellular glutamate fluorescence. Data were presented as mean ± standard deviation (SD). One-way ANOVA with Tukey’s post hoc test for multiple comparisons. ^**^p < 0.01, and ^***^p < 0.001 indicate statistical significance for comparisons between 5 Hz and 50 Hz. **F-H** TEM images of clear vesicles around synapses in cultured neuron cells without electrical stimulation (**F**) and after 5 min from electrical stimulation under 5 Hz (**G**) and 50 Hz (**H**). Scale bars: 100 nm. **I** The amount of vesicles from neuron cells after different electrical stimulation. Data were presented as mean ± standard deviation (SD). One-way ANOVA with Tukey’s post hoc test for multiple comparisons. ^*^p < 0.05, ^**^p < 0.01, and ^***^p < 0.001 indicate statistical significance for comparisons between 5 Hz and 50 Hz. **J** The size of vesicles from neuron cells after different electrical stimulation. Data were presented as mean ± standard deviation (SD). One-way ANOVA with Tukey’s post hoc test for multiple comparisons. ^*^p < 0.05, ^**^p < 0.01, and ^***^p < 0.001 indicate statistical significance compared with control. **K** Glutamate release from neuron cells cultured in the glutamate-deplete medium after different electrical stimulations. Data were presented as mean ± standard deviation (SD). One-way ANOVA with Tukey’s post hoc test for multiple comparisons. ^**^p < 0.01, and ***p < 0.001 indicate statistical significance compared with control group. **L** Glutamate uptake into glioblastoma cells under 0.05 to 50 µM concentrations of glutamate-based L-DMEM + P/S medium. Data were presented as mean ± standard deviation (SD). One-way ANOVA with Tukey’s post hoc test for comparisons. ^***^p < 0.001 indicates statistical significance. n.s., not significant (*p* > 0.05). **M** The cell viability of glioblastoma cells treated with different glutamate concentrations ranging from 0 to 100 µM based on two types of glutamate-deplete medium for 5 days. The value of 1 day in each concentration is considered 100% respectively. Data were presented as mean ± standard deviation (SD). One-way ANOVA with Tukey’s post hoc test for multiple comparisons. ^*^p < 0.05, ^**^p < 0.01, and ^***^p < 0.001 indicate statistical significance compared with day 1

To elucidate the impact of the neuronal microenvironment on glioblastoma proliferation, we investigated the transfer of neuronal secretions, such as neurotransmitters and neurotrophic factors, via their communication channels. We focused on glutamate, a key neurotransmitter that influences cellular growth and progression, which is abundantly present in the brain. The exocytotic secretion of glutamate from neurons upon exogenous stimulation has also been well-documented [39–42]. We postulated that varying levels of neuronal network excitability can regulate the secretion of neuronal factors influencing glioblastoma cell proliferation rates, and assessed using a protocol described in Fig. 3A. Pseudo-colored images obtained post-electrical stimulation indicate consistently higher intracellular glutamate synthesis at 50 Hz compared to unstimulated and 5 Hz-stimulated networks (Fig. 3B-E; Figure S9A-B, Supporting Information). The 50 Hz showed fold increases of 1.82, 1.32, and 1.27 compared to the 5 Hz at individual time points. Furthermore, glutamate synthesis increased by approximately 3.13-fold and 4.13-fold at 5 and 50 Hz, respectively, compared to control. The synthesis levels were sustained up to 4 h post-stimulation, as indicated by a non-significant difference (*p* > 0.05) between 1 and 4 h, contrasting with the significant changes (*p* < 0.05) observed from 5 min to 1 h (not shown in the graph).

In an effort to analyze glutamate secretion potential from presynaptic terminals, transmission electron microscopy (TEM) was utilized to analyze vesicle biogenesis and maturation. Round-shaped vesicles were identified in TEM images (Fig. 3F-H; Figure S9C, Supporting Information), and the frequency-dependent trends in vesicle numbers and diameters, calculated from the images, are shown in Fig. 3I-J. Initially, the number of vesicles post-electrical stimulation appeared similar across frequencies: 22.0 ± 12.7 at 5 Hz and 24.0 ± 10.5 at 50 Hz. Note that, at 1 h post-stimulation, the count at 50 Hz peaked at 28.7 ± 12.2 vesicles, while counts at 5 Hz declined to 6.3 ± 4.3. The hyperexcited neuronal networks sustained vesicle production over 24 h, gaining access to a reserve or resting pool, typically reserved for high-intensity synaptic activity, in addition to the rapidly recycling pool [43, 44]. Our observations are consistent with the notion that vesicle-associated neurotransmitter transporters, such as vesicular glutamate transporters (VGLUTs), primarily utilize electrochemical potential gradients across vesicle membranes for neurotransmitter loading [45–47]. In contrast, transporters for neurotransmitters such as GABA (gamma-aminobutyric acid) and monoamines (dopamine, serotonin, norepinephrine) tend to rely more significantly on vesicular pH gradients as the driving force for effective neurotransmitter uptake into synaptic vesicles [48]– [49]. Vesicle size, typically ranging from 30 to 100 nm in diameter and often appearing in pairs or groups, is a distinguishing feature of glutamate-containing vesicles [50–53]. Our size measurements confirmed that post-stimulation vesicles, despite their increased size, still belong to the glutamate-containing pool (Fig. 3J; Figure S9D, Supporting Information).

In a glutamate-free medium (B-2), glutamate release post-stimulation was measured (Fig. 3K). One hour after 50 Hz stimulation, glutamate release reached 2.05 ± 0.68 fmol per cell, 4.27-fold and 1.95-fold higher than the control and 5 Hz conditions, respectively. These findings indicate continuous, high-yield intracellular glutamate synthesis and release at 50 Hz. We also investigated the release of neuroligin-3 (NLGN3), a neurotrophic factor, post-stimulation as it is known to affect cell maturation and synaptic function in a paracrine manner [10, 54, 55]. The NLGN3, as revealed by the brain RNA-sequence analysis of Barres and colleagues, is highly expressed in neurons and oligodendrocyte precursor cells [56]. However, in our study, no significant changes were observed, suggesting that primary factor from hyperstimulated neuronal networks influences glioblastoma cells was glutamate (Figure S11, Supporting Information). This is a conflict result with the study by Venkatesh [10], which showed the growth of high-grade glioma due to the secretion of NLGN3 from neurons activated through optogenetic control. As for the decomposition of the synaptic adhesion molecule NLGN3 protein, which undergoes activity-dependent cleavage and secretion by protease, only unclear mechanisms based on indirect evidence are known to date, suggesting that there may be other inducing factors not only neuronal excitability. In addition, there are numerous paracrine signaling besides brain-derived neurotrophic factor (BDNF) that can exert an influence on glioblastoma, so further studies are needed to reveal them in the future [57].

### Glutamate up-regulates proliferation of glioblastoma cells

Further experiments on U87MG glioblastoma cells demonstrated concentration-dependent glutamate uptake, with significant differences in uptake rates at varying concentrations: the glutamate uptake was remarkably higher at 50 µM (Fig. 3L). The result suggests that extracellular glutamate concentration regulates uptake in U87MG. Long-term cell viability assays were performed using two different types of medium to examine the effect of glutamate on cell proliferation (Fig. 3M). The results revealed a proliferation threshold, beyond which cell viability declined [58]. Thus, glutamate is a critical component of the glioblastoma microenvironment, influencing biological progression through concentration-mediated interactions.

### Neuronal networks promote glioblastoma progress in an activation-dependent manner

**Fig. 4:**
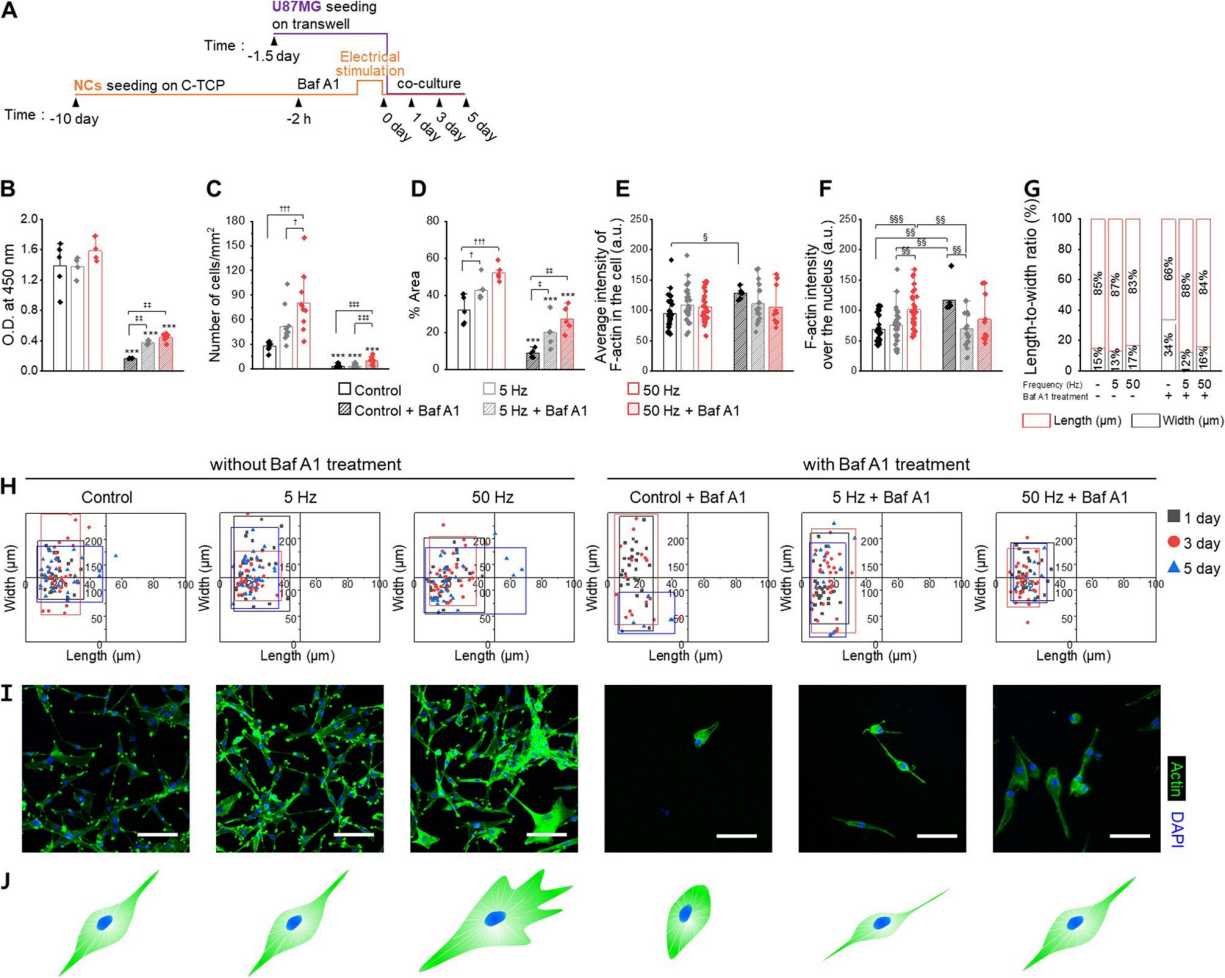
A hyperexcited neuronal network promotes glioblastoma progression in a glutamate-depleted medium. **A** Schematic description of co-culture experimental timeline for glioblastoma progression in a glutamate-depleted medium. Neuron cell culture parts are described as orange in color and glioblastoma cells are purple. Bafilomycin A1 (Baf A1) was used as a glutamate release blocker. **B-D** Cell viability (**B**), cell number (**C**), area coverage (**D**) of glioblastoma cells for 5 days. Data were presented as mean ± standard deviation (SD). One-way ANOVA with Tukey’s post hoc test for multiple comparisons. ^*^p < 0.05, ^**^p < 0.01, and ^***^p < 0.001 vs. without Baf A1 treatment of each group; ^†^*p* < 0.05, ^††^*p* < 0.01, and ^†††^*p* < 0.001 indicate statistical significance compared within without Baf A1 treatment group; ^‡^*p* < 0.05, ^‡‡^*p* < 0.01, and ^‡‡‡^*p* < 0.001 indicate statistical significance compared within with Baf A1 treatment group. **E**,** F** Average fluorescence intensity of actin filament in the cell (**E**) and actin filament intensity over the nucleus (**F**) of glioblastoma cells for 5 days. Data were presented as mean ± standard deviation (SD). One-way ANOVA with Tukey’s post hoc test for multiple comparisons. ^§^*p* < 0.05, ^§§^*p* < 0.01, and ^§§§^*p* < 0.001 indicate statistical significance compared within all group. **G** Stacked column of Length-to-width ratio of glioblastoma cells at day 5. **H** Scatter central plots showing the transition of width and length of glioblastoma cells during 5 days. **I**,** J** CLSM images (**I**) and illustrations (**J**) of representative morphology of co-cultured glioblastoma for day 5. Scale bars: 100 μm. Abbreviations: Control, without stimulation and + Baf A1, with Baf A1 treatment

To explore our hypothesis that glutamate secretion by neurons can modulate glioblastoma cell proliferation, we performed the in vitro co-culture of U87MG with neuronal networks after electrical stimulation in a glutamate-depleted medium. This approach was designed to isolate the influence of pathological neuronal responses on glioblastoma growth within a shared microenvironment while acknowledging the complexity of bidirectional interactions between the two cell types. The entire procedure, from electrical stimulation to co-culture, is summarized in Fig. 4A.

The changes in glioblastoma could be affected by incidental factors beyond glutamate. Therefore, before co-culture, glutamate release was assessed in the presence and absence of inhibitors, to ascertain the significance of glutamate in our co-culture model. Bafilomycin A1 (Baf A1) known as a powerful inhibitor of vacuolar ATPase suppressed the acidification of vesicles to block the transport of neurotransmitters and thus interrupted the cotransport of amino acid cross to the vesicle lumen from the cytosol. Our choice of Baf A1 as a glutamate inhibitor was based on the study conducted by Beltrán-Matas et al. on the hypothesis of preventing glutamate replenishment in synaptic vesicles [59, 60]. We determined the 2 h as the Baf A1 pre-treatment period for sufficient glutamate vacate in neuronal networks (Figure S12, Supporting Information).

The glioblastoma co-cultured with electrically stimulated neuronal networks demonstrated a frequency-dependent enhancement in metabolic activity, number of cells, and cell coverage area, which was evident even with Baf A1-treated groups (Fig. 4B-D; Figure S13-14, Supporting Information). Interestingly, despite clear increases in cell number and coverage area, the metabolic activity measured by the CCK-8 assay showed minimal changes at 50 Hz compared to control and 5 Hz groups. Microscope images revealed that glioblastoma cells under 50 Hz conditions formed dense clusters indicative of extensive proliferation. Cell coverage increased from 31.9 ± 7.6% (control) to 42.8 ± 6.3% (5 Hz) and 52.2 ± 4.3% (50 Hz) on day 5. The moderate increase between 5 and 50 Hz (1.25-fold vs. 1.55-fold) reflects this dense clustering behavior. We interpret the apparent discrepancy between metabolic activity (O.D. values) and the increased cell number and coverage as resulting from cellular clustering, leading to spatial limitations and potentially reduced oxygen and nutrient availability. Cells within clusters likely experience reduced metabolic activity without loss of viability. Thus, per-cell metabolic activity measurements may underestimate the true extent of cell proliferation under these conditions. Consistent with prior reports [61], this phenomenon shows how cell clustering can decouple metabolic activity from cell proliferation markers. Future work incorporating additional proliferation markers will further validate this interpretation. Exceptionally, the Baf A1-treated 50 Hz was observed an increase in metabolic activity and cell coverage area, along with a recovery of cell density on day 5. The results suggest that, without Baf A1 treatment, 50 Hz demonstrated a distinct advantage in glioblastoma growth, affirming the hypothesis that neuronal hyperstimulation promotes tumor growth. Additionally, the metabolic increase and cell number recovery observed in the 50 Hz with Baf A1 treatment imply the potential role of other paracrine factors, beyond glutamate, secreted from hyperexcited neuronal networks.

We also evaluated cell conditions using actin distribution according to the literature [62]. The average actin intensities across the cell body and over the nucleus were measured to estimate the distribution of actin fibers within cells and to characterize individual stress fibers (Fig. 4E-F). Impaired cells typically showed reduced F-actin intensity over the nucleus relative to the cell average, indicating a loss in stress fiber formation and chromatin condensation at the dendrite site, as observed in the groups co-cultured with neurons without stimulation and at 5 Hz. On the contrary, healthy cells, such as those in the 50 Hz, exhibited uniform actin fiber distribution, including over the nucleus. The Baf A1-treated control group showed a significant increase in average intensity and intensity over the nucleus, indicating the cell’s shrinking due to the cell death process. Baf A1-treated 5 Hz implied condensed F-actin on the membrane by progress of transformation into a spindle-shaped phenotype by showing a higher average intensity than intensity over the nucleus. In contrast, Baf A1-treated 50 Hz showed values reaching 100 a.u overall compared to other groups, suggesting uniform actin distribution in cells.

Morphological integrity and the average cell size are critical indicators of cellular health and integrity and we interpreted by three types of graphs, confocal laser scattering microscope (CLSM) images, and their representative cell phenotypes (Fig. 4G-J; Figure S15, Supporting Information): a stacked column for cell width and length ratio, a bar graph of length and width, and a scattered graph that tracks the change in width and length through distribution. The consistent length-to-width ratio (13–14%) on day 1 post co-culture across the without Baf A1-treated group revealed that glioblastoma cells maintained a spindle-like morphology, and reconfirmed in bar graph by similar length and width values. Over time, control and at 5 Hz either retained their size, whereas 50 Hz could be observed as the increase of width value. The scatter plots for the control and 5 Hz displayed a rectangular dispersion pattern with a vertically elongated axis, and the 50 Hz exhibited a rectangular dispersion pattern with a horizontally elongated axis, suggesting distinct phenotypic changes. The Baf A1-treated group exhibited a lower width ratio (9–13%) on day 1 than the untreated group. A significant change in the width ratio over time in Baf A1-treated control was observed, which is attributed to a substantial decrease in length due to cell shrinkage. This was further supported by the scatter plot, where the data points clustered in the third quadrant on day 5. The Baf A1-treated 5 Hz displayed a consistent ratio and plot distribution, exhibiting a more elongated spindle-like cell morphology indicating cellular stress. The Baf A1-treated 50 Hz exhibited an increase in width ratio throughout the experimental period, with gradually shifting towards the upper right of the plot dispersion area, indicating a recovery in morphology.

All results suggest that neuronal network activity influences glioblastoma cell proliferation through paracrine signaling even without direct cell-cell contact. Hyperactivated neuronal networks promote significant glutamate release, influencing glioblastoma survival and growth under nutrient-limited conditions. To confirm the robustness and generalizability of our observations, we expanded our experiments to include a second glioblastoma cell line, murine-derived GL261 cells (Figure S16, Supporting Information). Similar to U87MG cells, GL261 cells exhibited frequency-dependent changes in metabolic activity: control and 5 Hz groups initially increased metabolism but eventually declined significantly by day 5, while the 50 Hz group maintained elevated metabolic activity throughout. Furthermore, pretreatment with Baf A1 substantially suppressed metabolic activity, reinforcing glutamate’s key role. Neuronal activation partially overcame Baf A1-induced metabolic suppression, suggesting the possible involvement of additional neuron-derived factors beyond glutamate. These results support our conclusion that neuron-glioblastoma paracrine interactions significantly modulate glioblastoma proliferation.

### Hyperexcited neuronal networks encourage recurrence of glioblastoma against chemotherapy

**Fig. 5:**
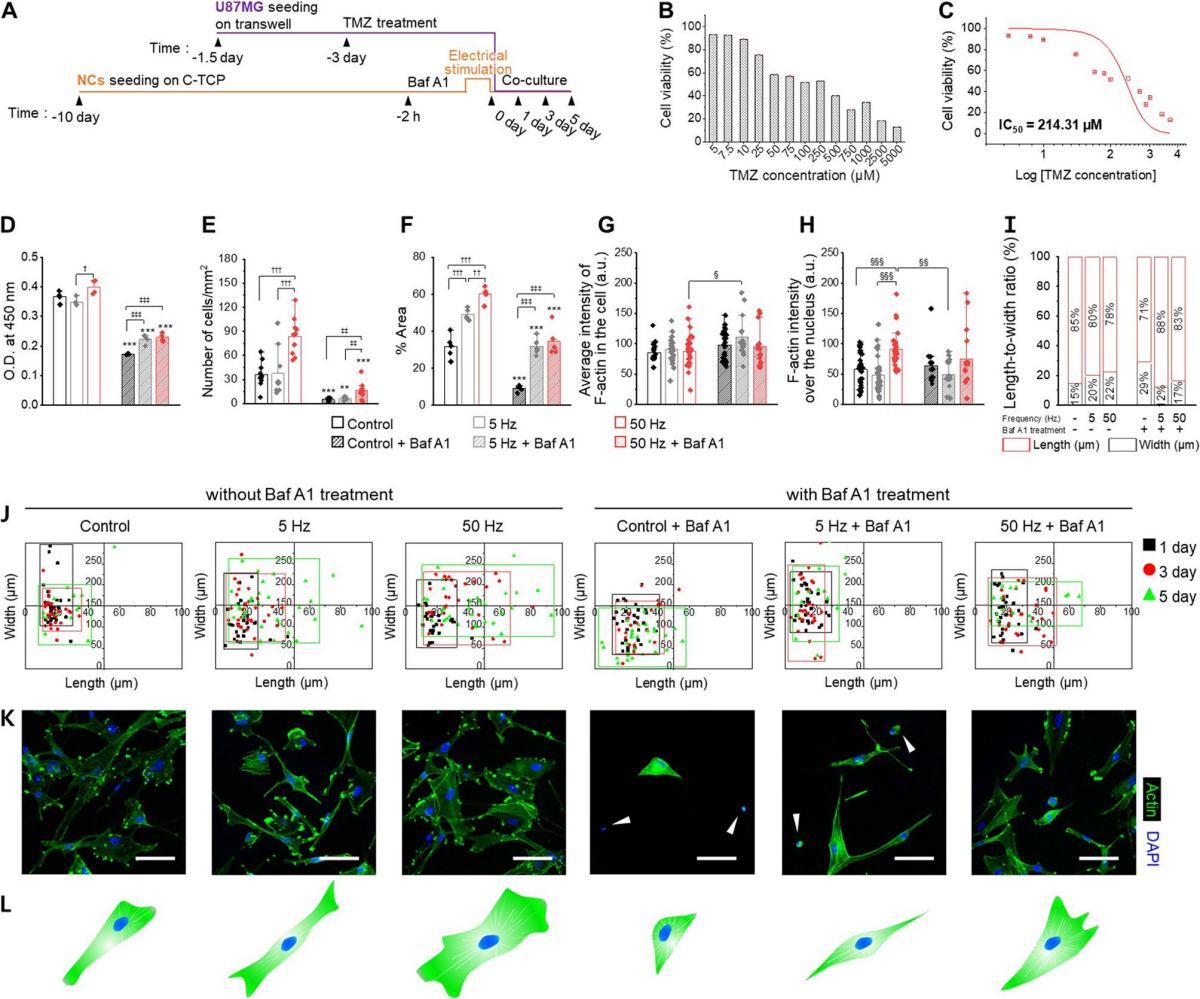
A hyperexcited neuronal network recurs glioblastoma after chemotherapy. **A** Schematic description of co-culture experimental timeline for glioblastoma recurrence after TMZ treatment. Neuron cell cultivation parts are described as orange in color and glioblastoma cells are purple. **B**,** C** Cell viability (**B**) and log IC_50_ calculation (**C**) of glioblastoma cells after 72 h TMZ treatment. Data were presented as mean ± standard deviation (SD). **D-F** Cell recurrence (**D**), number of cells (**E**), area coverage (**F**) of glioblastoma cells for 5 days. Data were presented as mean ± standard deviation (SD). One-way ANOVA with Tukey’s post hoc test for multiple comparisons. ^*^p < 0.05, ^**^p < 0.01, and ^***^p < 0.001 vs. without Baf A1 treatment of each group; ^†^*p* < 0.05, ^††^*p* < 0.01, and ^†††^*p* < 0.001 indicate statistical significance compared within without Baf A1 treatment group; ^‡^*p* < 0.05, ^‡‡^*p* < 0.01, and ^‡‡‡^*p* < 0.001 indicate statistical significance compared within with Baf A1 treatment group. **G**,** H** Average fluorescence intensity of actin filament in cell (**G**) and actin filament intensity over the nucleus (**H**) of glioblastoma cells for 5 days. Data were presented as mean ± standard deviation (SD). One-way ANOVA with Tukey’s post hoc test for multiple comparisons. ^§^*p* < 0.05, ^§§^*p* < 0.01, and ^§§§^*p* < 0.001 indicate statistical significance compared within all group. **I** Stacked column of Length-to-width ratio of glioblastoma cells at day 5. **J** Scatter central plots showing the transition of width and length of glioblastoma cells during 5 cultivation days. **K**,** L** CLSM images (**K**) and illustrations (**L**) of representative morphology of co-cultured glioblastoma for day 5. Scale bars: 100 μm. Abbreviations: Control, without stimulation and + Baf A1, with Baf A1 treatment

We investigated the role of paracrine signaling from the neuronal networks on glioblastoma sensitivity post-chemotherapy treated with temozolomide (TMZ) as described in Fig. 5A. TMZ, a standard treatment for glioblastoma, was evaluated for its role in potential recurrence (Fig. 5B-C) [63]. The cell viability of U87MG was measured across TMZ concentrations. The half-maximal inhibitory concentration (IC_50_) of TMZ for U87MG cells in our study was determined to be approximately 214 µM, which is consistent with previous in vitro reports using this cell line [64]. We acknowledge that this concentration is higher than clinically achievable levels in the cerebrospinal fluid, which are typically reported in the range of 1–10 µM. This discrepancy reflects the inherent TMZ resistance of U87MG cells and the absence of pharmacokinetic factors such as drug metabolism, blood–brain barrier transport, and protein binding in simplified in vitro systems. Our objective was not to replicate in vivo drug exposure levels, but to establish a controlled platform for evaluating relative changes in drug sensitivity, particularly in the context of neuronal influence following ELF stimulation. The use of a standardized high-dose exposure model facilitates reproducibility and allows for the detection of subtle modulatory effects, such as recurrence potential or resistance reversal, in a quantifiable manner. This approach has been widely adopted in glioblastoma chemoresistance studies using established cell lines.

Cellular metabolism, density, and coverage area were conducted to evaluate glioblastoma recurrence potential. When co-cultured with neurons without Baf A1 treatment, TMZ-treated glioblastoma cells exhibited metabolic recovery and a marginal increase in cell numbers, indicating the challenges of achieving complete remission only with chemotherapy (Fig. 5D-E; Figure S17, Supporting Information). Note that only glioblastomas in a hyperexcited neuronal network state demonstrated significant recurrence, characterized by both metabolic increase and cell number growth over time. The cell coverage area exhibited the highest value at 50 Hz, demonstrating a significant peak compared to other frequencies, and a clustering phenomenon, which was not observed in other groups, became apparent with the increase in cell numbers (Fig. 5F; Figure S18, Supporting Information). In contrast, Baf A1-treated 50 Hz displayed a significant metabolic increase without a corresponding rise in cell numbers over time, suggesting that the stimulus primarily impacted functional recovery rather than proliferation. Without stimulation, negligible metabolic change and consistently low cell counts were observed. Although a slight metabolic uptick was observed in the group stimulated at 5 Hz, a significant decrease in cell numbers suggests an unlikely recovery. These findings highlight the limited capacity for chemotherapy-treated cells to recuperate.

Actin intensity comparisons between cells and nuclei (Fig. 5G-H) showed stable states with average values approaching 100 a.u. in the 50 Hz-stimulated groups while other groups exhibited distinctly lower F-actin intensity over the nucleus. From the morphological analysis (Fig. 5I-L; Figure S19, Supporting Information**)**, the 50 Hz exhibited the highest rate of increase in width among the group without Baf A1 treatment, and the shift in the distribution of the plot towards the first quadrant over time suggests the potential for strengthening the thickness of glioblastoma processes following chemotherapy. In the Baf A1-treated group, the control cells exhibited a similar length-to-width ratio, consistent with cell shrinkage associated with cell death, and the data points were confined to the third quadrant in scatter central plots. In the 5 Hz, the plot distribution was observed in both the second and third quadrants, with the CLSM images revealing a mixture of very thin, spindle-shaped unhealthy cells and dead cells (Fig. 5K, white arrowheads). However, in the 50 Hz, some of the data points were distributed in the first quadrant on day 5, and the CLSM images showed cells with a relatively healthier phenotype, suggesting signs of recovery associated with hyperexcited neurons. This supports the hypothesis that neuronal hyperexcitation is functionally relevant to glioblastoma recurrence post-chemotherapy.

## Discussion

The phenomenon of glioblastoma recurrence leading to death after chemotherapy in neuropathology patients with abnormal physiological signals from neuronal networks was identified in clinical research, but the study for neuron-to-tumor interaction in the brain remains at the early research level. Given the substantial influence of electric fields on neuronal activities, neuron-glioma communication may serve as a vital mechanism for the activation of brain cancer cells by environmental electric fields. Here, we identified proof of concept at the microenvironment scale via a customized in vitro setup that the phenomenon of glioblastoma from proliferation to recurrence which affects mortality in patients after chemotherapy is led by hyperexcited neuronal activity. Neuronal activation is stimulated in a circulation manner according to the structural characteristics of the network, and stimulation originating from an axon at one location propagates in both directions, causing new stimulation in each branch and synergistic firing to finer branches [65]. Changes in the activity of these synapses are generally direct conversions of changes in electrical potential, and the main element is the electrical signals used to transmit information between nerve cells. In neurodynamics, the firing pattern of nerve cells, which is the starting point of physiological signal patterns, is divided into three main types, and the importance of changes in the pattern according to the initial explosion is recognized and strives to reproduce through neuron model as the adaptive exponential integrate-and-fire model (AdEx model) [66, 67]. In 2005, Romain Brette and Wulfram Gerstner showed the reproduction of the regular-spiking Hodgkin-Huxley type model through simple electrophysiological protocols, current pulses, steps, and ramps, and suggested the possibility of fitting it to real neurons according to electrical stimulation [68].

Based on this, we were also able to successfully emulate three neural network models based on electrical stimulation: normal state (no stimulation), awake-like state (5 Hz), and abnormal state (50 Hz). It should be acknowledged that the electric field strength used in this study (100 V m^−1^) and the short stimulation duration (5 min) exceed the magnitude and duration of ELF fields typically encountered in everyday human environments [13, 14]. According to WHO and ICNIRP guidelines, environmental ELF electric fields are generally < 10 V m^−1^ in residential settings and up to 5 kV m^−1^ near high-voltage transmission lines, with basic restrictions for internal brain fields as low as 0.8 V m^−1^ at 50–60 Hz [11, 69]. Nonetheless, the primary aim of our study was not to replicate environmental exposure per se, but to model the biological consequences of neuronal hyperexcitation induced by ELF-range stimuli. Previous in vitro studies have demonstrated that neuronal activation, including calcium influx and glutamate release, can be effectively induced by field strengths in the range of 50–150 V m^−1^ without causing cytotoxicity. These intensities provide a practical and biologically relevant threshold to trigger activity-dependent paracrine signaling. Moreover, modeling low-level chronic ELF exposure in vitro is inherently constrained by limitations in maintaining long-term field homogeneity, temperature, and neuronal viability. As such, our stimulation paradigm, while not directly mimicking real-world exposure, serves as a mechanistic proxy to probe how sustained or intense neuronal activation may influence glioblastoma behavior. We therefore clarify that our results do not imply a direct health risk from ambient ELF fields, but instead provide a proof-of-concept model that could inform future studies exploring the impact of sustained neurophysiological disturbances, whether environmentally induced or iatrogenic, on tumor recurrence within the brain microenvironment.

In this study, we employed primary hippocampal neurons as the excitatory network model due to their well-established use in in vitro systems and their capacity to form reproducible, synaptically connected glutamatergic networks with minimal non-neuronal contamination. These neurons exhibit robust responses to electrical stimulation, making them suitable for analyzing activity-dependent glutamate release and its downstream effects on tumor cells. However, we acknowledge that hippocampal neurons do not fully recapitulate the physiological properties of cortical neurons, which are more directly implicated in the peritumoral environment of glioblastoma. Cortical neurons display distinct patterns of intrinsic excitability, synaptic transmission dynamics, and glutamate homeostasis. In particular, regional differences in the expression of glutamate transporters (e.g., EAAT1/2) and vesicular glutamate release properties may influence the amplitude and spatial distribution of paracrine signaling to glioma cells [70–72]. These variations could impact the extent to which neuronal activity modulates tumor growth or recurrence in vivo. While our findings provide a mechanistic framework for neuron-to-glioma signaling under ELF stimulation, future studies should incorporate region-specific neuronal subtypes, such as primary cortical neurons or human iPSC-derived cortical neurons, to better simulate the peritumoral microenvironment and assess the anatomical specificity of the observed interactions.

Abnormal changes in burst firing subtypes according to nervous system activity suggest the involvement of stress or the pathology of neurological diseases such as anxiety, depression, epilepsy, and seizures, and the significance of the correlation between specific neural activity following burst firing and tumor progression has particular implications in the clinic [28, 73–76]. Unfortunately, to date, the interconnection of neuron-tumor networks remains unclear, raising the need to understand the fundamental principles that contribute to disease progression through the implementation of the tumor microenvironment. In particular, the progress of studies showing the effects on nerves as the disease progresses [7, 8, 54, 57, 63] and, paradoxically, the absence of studies that analyze neural activation in detail and reveal the related interactions, suggest we have missed out on insight into new treatments. Accordingly, our customized in vitro model, which enables co-culture of gliomas in independent spaces under an activation level-dependent neural network, serves as an advanced tool for predicting complex interactions within a shared microenvironment and allows for imaging analysis feasible. Still, our co-culture model does have significant limitations, including the absence of direct physical connections between cells, precluding juxtacrine signaling, and it does not fully represent the intricate multicellular interplay found in three-dimensional glioblastoma biology. Therefore, there is a compelling need for further studies to develop more comprehensive systems that address these biological gaps.

Cooperation between the neural network and glioblastoma relies on secretory signal transmission depending on the level of neural network activation was confirmed in two stages, monoculture and co-culture. The series of processes including glutamate transfer to the nerve cell terminal synapse, capture through vesicles, and its extracellular release showed that physiological signal changes of the neuron network as regulators, and endocytosis and viability up-regulation to glioblastoma via glutamate provides a stepping stone to the next coculture experiment. Of note, it is important to note that the measured concentration of glutamate in the bulk medium (~ 5 µM) following neuronal stimulation appears lower than the threshold (~ 50 µM) required to induce robust proliferation effects in glioblastoma cells under monoculture conditions. However, this apparent discrepancy may be attributed to the formation of localized pericellular glutamate microdomains near the transwell membrane [77–79]. Glioblastoma cells are cultured on the transwell insert, positioned in close proximity to the neuronal monolayer beneath, where glutamate release occurs. Under such spatial arrangements, diffusion constraints and sustained synaptic activity may generate transiently elevated glutamate concentrations at the cell surface that are not reflected in bulk media measurements. This is consistent with previous reports demonstrating steep concentration gradients across microporous membranes in transwell systems and synaptic models [80]. Furthermore, our data indicate that even moderate glutamate levels (5–10 µM) can elicit measurable effects on glioblastoma cell viability over prolonged exposure, suggesting a non-linear dose-response relationship. To more directly evaluate the hypothesis of localized glutamate enrichment, we have initiated the development of a FRET-based glutamate biosensor localized on the glioblastoma cell membrane. This approach will enable real-time monitoring of pericellular glutamate concentrations and their dynamics during co-culture with stimulated neurons. Such direct measurements will help validate whether spatially confined glutamate exposure contributes to the proliferative response observed in our model.

We used Baf A1 as a pharmacological tool to inhibit glutamate release from neuronal networks. Baf A1 effectively blocks vacuolar-type H+-ATPases, thereby disrupting the proton gradient required for vesicular glutamate loading. However, we acknowledge that this inhibitor is non-specific and acts globally across various intracellular compartments, including endosomes, lysosomes, and multiple vesicle types. Such broad activity may influence other aspects of neuronal physiology and glioblastoma cell behavior beyond glutamate signaling, introducing potential confounding effects. To more specifically validate the role of glutamate in mediating neuron-to-glioma communication, future studies will employ targeted approaches. For instance, neuron-specific knockdown of vesicular glutamate transporters (e.g., VGLUT1) via siRNA or CRISPR interference would allow selective inhibition of glutamate packaging without disrupting other neurotransmitters. Additionally, small-molecule VGLUT inhibitors such as Rose Bengal provide a pharmacological route to suppress glutamate release with higher specificity. These complementary strategies will help dissect the specific contribution of glutamate within the broader context of neuronal secretome and enhance the mechanistic resolution of neuron-induced glioblastoma proliferation.

Although glutamate emerged as the most prominent factor secreted from hyperexcited neurons in our model, it is unlikely to act in isolation. Neuronal activation is known to trigger the release of a broad repertoire of neuroactive molecules, and it is plausible that these co-released factors collectively contribute to the observed glioblastoma responses. For example, brain-derived neurotrophic factor (BDNF) is secreted in an activity-dependent manner and has been shown to enhance glioma cell survival, motility, and angiogenic potential through TrkB–ERK–VEGF signaling pathways [57, 81, 82]. Similarly, extracellular ATP, released during neuronal depolarization via pannexin/connexin hemichannels, can stimulate purinergic receptors on glioblastoma cells, influencing intracellular calcium dynamics and pro-tumorigenic signaling [83–85]. Moreover, neuron-derived cytokines such as IL-6 and chemokines like CXCL12 have been implicated in promoting glioma proliferation, immune evasion, and stem-like cell recruitment [86]– [87]. In our study, we focused on glutamate due to its well-characterized role in neuron–tumor interactions and its reliable detection using enzymatic assays. However, we recognize that the current findings do not exclude the involvement of other soluble mediators. Future work will incorporate multiplexed secretome profiling, transcriptomic analysis of glioma responses, and the use of receptor-specific antagonists to evaluate the combined influence of neurotrophic and inflammatory signals in this context. Recognizing these additional pathways broadens the interpretative scope of our findings and reveals the complexity of the neuron–glioma paracrine network under electrophysiological stimulation.

Proliferation and post-chemotherapy recurrence through the co-culture system using a medium that eliminates glutamate to maximize the impact of paracrine signals from neuronal networks via stimulation, in a parallel manner, more clearly demonstrate the influence of activated neural networks on glioblastoma as seen in previous monoculture studies. In promoting cell proliferation and recovering damaged cell characteristics, neurological diseases and stress can predict the rapid progression of early-stage glioblastoma patients or the recurrence of glioblastoma patients who have completed chemotherapy, even if only by leaps and bounds. However, current research lacks an understanding of the neurotransmitters and neurotrophic factors mediated by the neural network microenvironment rather than glutamate, and the structural and cell composition that consists of the microenvironment are simple, requiring additional research and various types of models. While this study provides mechanistic insight into the role of neuron-derived paracrine signaling in glioblastoma recurrence following chemotherapy, it is important to acknowledge that the findings are limited to an in vitro setting. Our experimental design intentionally employed a simplified co-culture model to isolate the contribution of electrically stimulated neuronal networks to GBM cell behavior, excluding systemic, vascular, and immune components. However, for clinical translation and validation, in vivo models that recapitulate the tumor–brain microenvironment will be essential.

Future work will aim to implement orthotopic xenograft models in immunodeficient mice bearing human glioblastoma tumors to evaluate whether ELF exposure, either directly or through activation of host neuronal circuits, promotes tumor recurrence after temozolomide treatment. Such studies will require precisely controlled delivery of low-frequency electromagnetic fields within the brain while minimizing confounding thermal or systemic effects. Furthermore, dissecting the neuronal contribution in vivo will necessitate the use of cell-type-specific genetic tools such as inhibitory DREADDs (e.g., hM4Di) to selectively silence neuronal activity and confirm its role in modulating tumor progression [88–90]. Although technically challenging, these approaches represent a logical extension of our current work and will be critical to establishing the physiological relevance of ELF-induced neuron–tumor crosstalk. We believe that our in vitro model provides a necessary foundation for developing such in vivo systems, enabling precise dissection of microenvironmental mechanisms underlying GBM recurrence. The findings presented here reveal the unexplored potential for the treatment of patients through a better understanding of the impact of neural networks on incurable glioblastoma.

## Materials and methods

### Experimental design

The in vitro experimental setup apparatus was designed and fabricated as described in Fig. 1, A to C. A coverslip (12 mm in diameter) was fixed with 100 µL polydimethylsiloxane elastomer (PDMS, B41_000009, Dow Corning, Seoul, Republic of Korea) on a 24-well tissue culture plate (TCP) for further immunocytochemical staining and cell imaging after cultivation. Customized platinum (Pt) electrodes with the design selected by COMSOL Multiphysics^®^ simulation were placed in intervals of 10 mm on both sides of C-TCP without direct contact with the coverslip. The applied alternating current (AC) wave was run in paired Pt-electrodes at 5 and 50 Hz under 1 V for 5 min modulated by an Arbitrary function generator (AFG31022, Tektronix, Oregon, US).

### Computational modeling for electrical stimulation

The electrode model inserted in a medium within a 24-well tissue culture plate was developed using COMSOL Multiphysics^®^ software (COMSOL, Inc., Stockholm, Sweden) to investigate an electric field distribution and electric potential for the preparation of electrodes. The requirements for the electrodes were length and thickness definition. The gap between the Pt-electrodes is 10 mm for a sufficient field of electrical stimulation to accommodate multiple cells. The AC/DC module of the COMSOL Multiphysics^®^ software has been selected. We adjust the relative permittivity parameters for developing the COMSOL model as follows: water relative permittivity (78) and platinum relative permittivity (2.6) [91]. A physics-controlled mesh with a normal element size was employed to balance computational efficiency and spatial resolution. The solver convergence criterion was defined by setting the relative tolerance to 0.01, ensuring that the solution converged when the residual norm was reduced below 1% of its initial value. The electrode length and thickness were changed during simulation from 5.0 to 12.0 mm and from 0.01 to 1.00 mm to determine the optimal value. The entire domain was modeled using tetrahedral elements, and the charge conservation physics interface was applied with electrical insulation imposed on all external boundaries. Electric potential (1 V) and ground (0 V) were assigned to the respective electrode boundaries, establishing a unidirectional electric field that allowed precise analysis of potential and field distribution within the domain. The simulation results of only one pair representing ideal values are adopted in the study.

### Primary hippocampal neuron cultures and glioblastoma cell lines

Primary hippocampal neurons were prepared from an SD rat (18 days gestation, Koatech, Pyeongtaek, Republic of Korea) as previously described [22] and summarized in Figure S2A (Supporting Information). The protocol for the animal studies was approved by the KAIST Institutional Animal Care and Use Committee (KA2023-049). Briefly, hippocampi were isolated from the rat pups, digested with trypsin solution at 37 °C for 15 min, and dissociated. Neuron cells (NCs) were seeded at a density of 1.2 × 10^5^ cells/well on C-TCP pre-treated with poly-D-lysine hydrobromide (PDL, P6407, Sigma Aldrich, St. Louis, MO, USA) and laminin (23017015, Gibco, Thermo Fisher Scientific, Waltham, MA, USA). NCs were initially maintained in a plating medium consisting of Neurobasal medium (NM, 21103-049, Gibco) containing 10 µL mL^−1^ 100× Glutamax™ Supplement (35050079, Gibco), 10 µL mL^−1^ 50× B-27TM Supplement (B27, 17504-044, Gibco), 10 µL mL^−1^ penicillin and streptomycin (P/S, 15140-122, Gibco), and 0.125 µL mL^−1^ 10 mM L-glutamic acid (Sigma Aldrich, St. Louis, MO, USA). Afterward, half of the medium was replenished twice a week with a culture medium consisting of NM containing 10 µL mL^−1^ Glutamax, 10 µL mL^−1^ B27, and 10 µL mL^−1^ P/S, and NCs were used for study after 10 days-in-vitro (DIV).

For monocultures of glioblastoma cell line obtained from the Korean cell line bank (U87MG, Seoul, Republic of Korea) was seeded at a density of 2.0 × 10^3^ cells per well and cultured in high-glucose Dulbecco’s Modified Eagle Medium (DMEM, Gibco) supplemented with 100 µL mL-1 fetal bovine serum (FBS, Gibco), and 10 µL mL^−1^ P/S and placed in a standard humidified incubator at 37 °C and 5% CO_2_ air atmosphere. U87MG was used for experiments 36 h after plating at which time they were in a stable state.

NCs cultured DIV 10 and U87MG cultured for 36 h were replaced with fresh medium listed in Table S1. Cells were then cultured with different mediums, and the cell viability and their morphologies were analyzed for 5 days.

### Cytotoxicity/viability, nuclei counting, cell coverage area, and cell adhesion

Cell cytotoxicity/viability analysis was determined by cell counting-8 assay (CCK-8; CK04, Dojindo, Kumamoto, Japan). After refreshing to a fresh medium, 10 v/v-% of CCK-8 was added to each well and incubated for 2 h. The 100 µL of the resulting solution was transferred to a 96-well plate, and the absorbance of each well was obtained using a microplate reader (CLARIOstar, BMG Labtech, Ortenberg, Germany) at 450 nm.

For cell counting experiments, cells were fixed in 4% paraformaldehyde (Para, J61899.AP, Alfa Aesar) at 4 °C. Cell specimens were permeabilized by Triton X-100 (T9284, Sigma Aldrich, St. Louis, MO, USA) for 5 min and blocked by 1% bovine serum albumin (BSA, A2153, Sigma Aldrich, St. Louis, MO, USA) for 20 min. All specimens were mounted by a Vectashield mounting medium for fluorescence with DAPI (H-1200, Vector Laboratories. Inc, Newark, CA, USA) on confocal dishes (101350, SPL, Pocheon, Republic of Korea). Imaging was performed using a confocal laser scanning microscope (CLSM; LSM880, Carl Zeiss, Oberkochen, Germany‎). The analysis of images was automated as follows: thresholded to the same value, inverted, and selected five areas for unbiased quantification using Image J software with the Fiji function named analyze particles (Figure S2B-C, Supporting Information). The cell-covered area was quantified using images from a microscope (DMI 3000B, Leica, USA). The images were automated as follows: thresholded to the same value, inverted, and selected five areas for unbiased quantification using Image J software with the Fiji function measuring % area. The cell adhesion efficiency was analyzed using a counting plate. After 24 h of cell seeding, we collected the supernatant and dripped it onto the counter. According to the calculation of the cells in the counting plate, counting only intact cells. After counting, the number of cells per seeding density was calculated.

### Electrical stimulation of cells

The NCs cultured on C-TCP in DIV 10 were substituted with a glutamate-depleted medium. Two electrodes were fixed on both sides of the round C-TCP and connected to the function generator. This device was able to give AC electrical stimulation in the sin curve to cells on the C-TCP. The stimulation parameters were 1.0 V of electrical strength, 5 min of electrical time, and 5 and 50 Hz of frequencies as shown in Figure S5-7 (Supporting Information).

### Bafilomycin A1 treatment for glutamate inhibition

Bafilomycin A1 (Baf A1, Sigma Aldrich, 10 µg) prepared in dimethyl sulfoxide (DMSO, D8418, Sigma Aldrich, 100 µL) was diluted as 1 µM in a glutamate-depleted medium for accelerating inhibition effect supported by replenishment. DIV 10 neuronal cultures were pretreated with 1 µM Baf A1 for 2 h to inhibit glutamate loading into synaptic vesicles, and subsequently washed three times with fresh glutamate-depleted medium prior to electrical stimulation and co-culture with GBM cells.

### Calcium imaging

Calcium transients of the intracellular network were monitored by calcium indicator dye Fluo-4 AM (F14201, Thermo Fisher Scientific). The NCs were loaded with the 200 µL mixture of 1 µL Fluo-4 AM, 10 µL Power Load TM (P10020, Thermo Fisher Scientific), and 989 µL Hanks’ Balanced Salt solution (HBSS, 14175-079, Gibco) for 30 min at 37 °C after washing by HBSS. The electrical stimulation was proceeded and immediately washed 2 times with HBSS and the fluorescence signal was observed on a fluorescence microscope (DMI 3000B, Leica, USA) equipped with a filter (blue, excitation range: bandpass 450–490 nm and emission range: long pass 515 nm). Calcium fluorescence images were first processed using ImageJ software with the Fiji distribution (NIH, MD, USA) to generate pseudo-color representations. ROIs were manually selected based on a predefined criterion—the presence of a clearly identifiable neuronal soma —in a blinded manner to minimize bias. All imaging parameters, including exposure time (250 ms), and detector gain (3.0), were kept constant across experimental groups. Fluorescence intensity values extracted from the ROIs were subsequently quantified using Origin2019 (OriginLab Corp., MA, USA) with uniform analysis settings. The analysis was performed using the following workflow: *Analysis > Peak and Baseline > Peak Analyzer > Integrate Peaks.* This standardized workflow was consistently applied across all samples to ensure uniform analysis parameters. Different types of calcium events such as neuronal firing and calcium ion shifting were manually selected and described in sequence. The quantitative data in calcium imaging analysis are selected from the response cells, therefore the number of dots for each group is variant (Fig. 2; Figure S5-7, Supporting Information).

### Sampling and detection of neurotransmitters

The quantitative amount of released/uptake glutamate into the medium from the cultures was measured by an Amplex™ Red Glutamic acid/Glutamate Oxidase Assay Kit (Thermo Fisher Scientific). For endogenous glutamate measurement, cultivated NCs in a complete culture medium for 10 DIV were substituted with the 500 µL of fresh glutamate-depleted medium and elicited by electrical stimulation. The supernatants were collected at 1, 3, 5, 9, 12, and 15 h. For glutamate uptake measurement, the supernatants were collected at 1, 3, 7, 10, 15, and 30 min from the cultivation medium in the presence of 0.05 to 50 µM glutamate. Glutamate concentration was quantified using an Amplex™ Red Glutamic acid/Glutamate Oxidase Assay Kit following the instructions of the manufacturer. The luminescence was measured by a microplate reader.

### Transmission electron microscopy

The cell specimens were washed and collected in ice-cold phosphate-buffered saline (PBS, 10010-049, Gibco) and centrifuged at 5,000 rpm for 3 min. Pelleted cells were fixed directly with 2% Para and 2% glutaraldehyde (G5882, Sigma Aldrich) in 0.05 M sodium cacodylate buffer overnight at 4 °C, washed three times in 0.5 M sodium cacodylate buffer, post-fixed using 1% osmium tetroxide in 0.05 M sodium cacodylate buffer at 4 °C for 1.5 h, and washed two times with distilled water. The cell specimens were en bloc stained in 0.5% uranyl acetate at 4 °C overnight, dehydrated through a graded series of ethanol mixture up to 100% at room temperature, and transited with 100% propylene oxide. The specimens were embedded in resin mixture in order as follows: 1:1 mix of propylene oxide and Embed 812 resin mixture for 2 h, 1:3 mix of propylene oxide and Embed 812 resin mixture for 4 h, and 100% Embed 812 resin mixture for 2 h. The specimens were transferred into fresh 100% Embed 812 resin mixture again and polymerized for 48 h at 60 °C. The specimens were sectioned around 70–80 μm thickness by ultramicrotome (UC7/FC7, Leica, Wetzlar, Germany), and the sections were collected on a 200-mesh copper EM grid, counterstained using uranyl acetate and lead citrate, and examined using transmission electron microscopy (TEM H-7650, Hitachi, Tokyo, Japan). Synaptic vesicles were manually identified and quantified using ImageJ software (NIH, USA). A fixed size threshold of 20–100 nm was applied for vesicle detection, and vesicle diameters were manually measured based on calibrated scale bars. Counting was performed on 2–8 randomly selected synaptic regions per specimen by two independent observers blinded to the experimental conditions to minimize observer bias.

### Immunocytochemistry

For evaluate the purity of hippocampus-derived primary neurons, cells were subjected to immunostaining using specific markers. In brief, the cells were fixed with 4% Para overnight, followed by permeabilization with 0.1% Triton X-100 for 5 min. Neuronal purity was assessed by staining by primary antibodies of rabbit-MAP2 (1:200) (4542 S, Cell signaling technology^®^, Danvers, MA, USA), and mouse-GFAP (1:200) (G3893, Sigma Aldrich), and secondary antibody of anti-mouse 488 Alexa Fluor (1:400) (Thermo Fisher Science) and anti-rabbit 546 Alexa Fluor (1:400) (Invitrogen), to distinguish neurons from glial cells.

For glutamate immunocytochemistry based on the description of the prior studies [92, 93], STAINperfrect Immunostaining Kit A (SP-A-1000, ImmuSmol, Bordeaux, France) was used according to the protocol of the manufacturer to detect L-glutamine. The primary antibodies used for NCs were mouse anti-L-Glutamate (1:500) (IS018, ImmuSmol, Bordeaux, France) and rabbit anti-β-tubulin III (1:500) (T2200, Sigma Aldrich), and secondary antibody for immunolabeling was anti-mouse 488 Alexa Fluor and anti-rabbit 546 Alexa Fluor, 1:1000 in Antibody Diluent (ImmuSmol).

For NLGN 3 immunocytochemistry staining steps based on the description of the previous execution [94], cell specimens fixed with 4% Para were blocked and permeabilization proceeded in 0.1% Triton X-100 for 5 min after washing with PBS. The cells were treated with primary antibodies of mouse anti-NLGN3 (1:100) (sc-50395, Santa Cruz Biotechnology, Texas, USA) and rabbit β-tubulin III (1:500), and secondary antibodies of anti-mouse 488 Alexa Fluor (1:400) and anti-rabbit 546 Alexa Fluor (1:1000) based on 1% BSA solution.

For visualizing U87MG morphology to determine whether the U87MG is affected by an electrically stimulated neuronal network during the co-culture, cell specimens were stained. After fixation of the cells with 4% Para, cell specimens were permeabilized with 0.1% Triton X-100 for 5 min. Afterward, samples were incubated with ActinGreen™ (R37110, Invitrogen) conjugated in 1% BSA for 20 min at room temperature.

All cell specimens were subsequently mounted on confocal dishes with a Vectashield mounting medium for fluorescence with DAPI that simultaneously counterstained nuclei. Images were taken using CLSM. All images were analyzed based on five randomly selected fields per image for quantitative analysis and images were further processed with Image J software with the Fiji function.

### ELISA

The NCs cultured for 10 DIV were substituted with the fresh medium and elicited the release of neuroligin-3 (NLGN3) by electrical stimulation. The supernatants were collected at 0.5, 5, 10, 15, 30, and 60 min. NLGN3 was quantified by ELISA following the instructions from the manufacturer. Rat Neuroligin-3 ELISA Kit (E2338Ra, BT LAB, Birmingham, England) was used, and the luminescence was read by a microplate reader at 450 nm.

### Dose determination study

Temozolomide (TMZ, Sigma Aldrich, 10 µg) was dissolved in DMSO (100 µL) and diluted in a culture medium. After 36 h from glioblastoma seeding, TMZ was added to each well to serial dilution of different dose concentrations, and treated cells were incubated for 72 h after which cell viability was assessed. A CCK-8 assay was performed to demonstrate the IC_50_ TMZ. The 200 µM of drug concentration chosen for this study was taken from calculation results.

### Co-culture experiment

In the case of glioblastoma behavior under a glutamate-depleted environment, the stimuli recipient NCs were seeded in C-TCP, as described above, and were used after DIV 10. U87MG and GL261 were seeded in Falcon^®^ Permeable Support for a 24-well plate with a 0.4 μm transparent PET membrane (transwell; Corning, New York, USA) at a density of 500 cells per well and incubated for 36 h at 37 °C in 5% CO_2_ incubator. NCs were treated with Baf A1 for 2 h before electrical stimulation, and the U87MG-seeded transwell was transferred to the upper chamber of C-TCP after stimuli of NCs in a fresh medium.

In the case of glioblastoma recurrence analysis after chemotherapy, U87MG cultivation was performed using a transwell in 500 cell/well seeding density. After incubation for 36 h, U87MG was treated with TMZ for 72 h and substituted with a fresh glutamate-depleted medium. The stimuli recipient NCs were cultured on C-TCP for DIV 10 and took Baf A1 treatment for 2 h before electrical stimulation. The transwell was transferred to the upper chamber of C-TCP after stimuli of NCs in a fresh medium for co-culture.

For co-cultures of U87MGs with NCs, the optimized media consisted of NM + B27 + P/S. The five groups depending on NC condition were as follows: without stimulation, 5 Hz electrical stimulation (5 Hz), 5 Hz electrical stimulation after Baf A1 treatment (5 Hz + Baf A1), 50 Hz electrical stimulation (50 Hz), and 50 Hz electrical stimulation after Baf A1 treatment (50 Hz + Baf A1).

### Statistical analysis

All experiments were performed independently using distinct biological replicates derived from separate cell preparations. Unless otherwise noted, each experiment was conducted with three independent biological replicates (*n* = 3). An exception was the Ca²⁺ spike analysis, for which *n* = 40 regions of interest (ROIs) were randomly selected from time-lapse recordings and analyzed as technical replicates. In cases where imaging-based quantification was involved, data analysis was conducted using automated software with uniformly applied parameter settings to ensure consistency and minimize operator bias. The values are expressed as mean ± standard deviation (SD). The statistical significance of the data was evaluated with one-way ANOVA followed by Tukey’s multiple comparison post hoc test for multiple comparisons. The criterion for statistical significance was denoted as follows: *p < 0.05, **p < 0.01, and ***p < 0.001. Abbreviation: n.s. is not significant, and N/D is not detectable.

## Supplementary Information

Supplementary Material 1.

## Data Availability

No datasets were generated or analysed during the current study.
