# Supplementary material for: Aberrant neuronal firing: a paracrine route to glioblastoma expansion
Source: Cell Commun Signal. 2025 Sep 25;23:398. doi: 10.1186/s12964-025-02404-8 (PMC12465965; doi:10.1186/s12964-025-02404-8)
Supplement: Supplementary file 1 — Supplementary Material 1. [file 12964_2025_2404_MOESM1_ESM.docx]

**Supplemental information**

**Aberrant neuronal firing: A paracrine route to glioblastoma expansion**

**Ji Yeon Lee, Bon Il Koo, Trang Huyen Le-Kim, Yoonsung Nam**


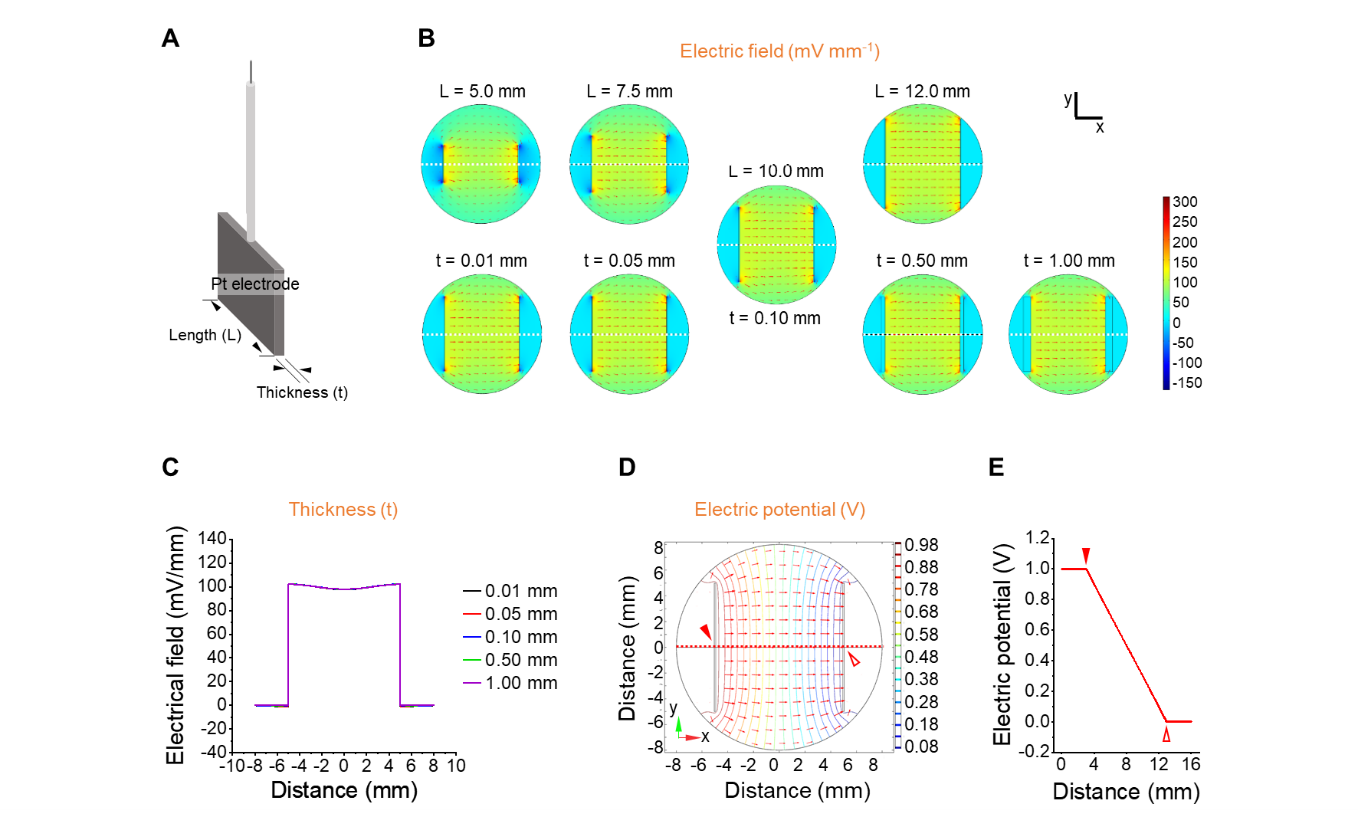


**Supplementary Figure S1:** **COMSOL Multiphysics modeling of Pt-electrode for optimum electrode configuration**. (A) A schematic of the Pt-electrode: Length (L) and thickness (t). (B) Comparison of electric field distribution delivered by different lengths (L; top) and thicknesses (t; bottom) of electrodes on stimuli by a finite element method using COMSOL Multiphysics software. The color-coding of the scale bar represents the range of values for the electric field (mV mm^-1^). (C) The electric field strength generated by electrodes with thickness from 0.01 to 1.00 mm. (D) The electric potential distribution on stimuli by electrodes with 10.0 mm length and 0.10 mm thickness. The color-coding of the scale bar represents the range of values for the electric potential (V). (E) a cross-section plot of electric potential along a horizontal line (red dotted line) in D.


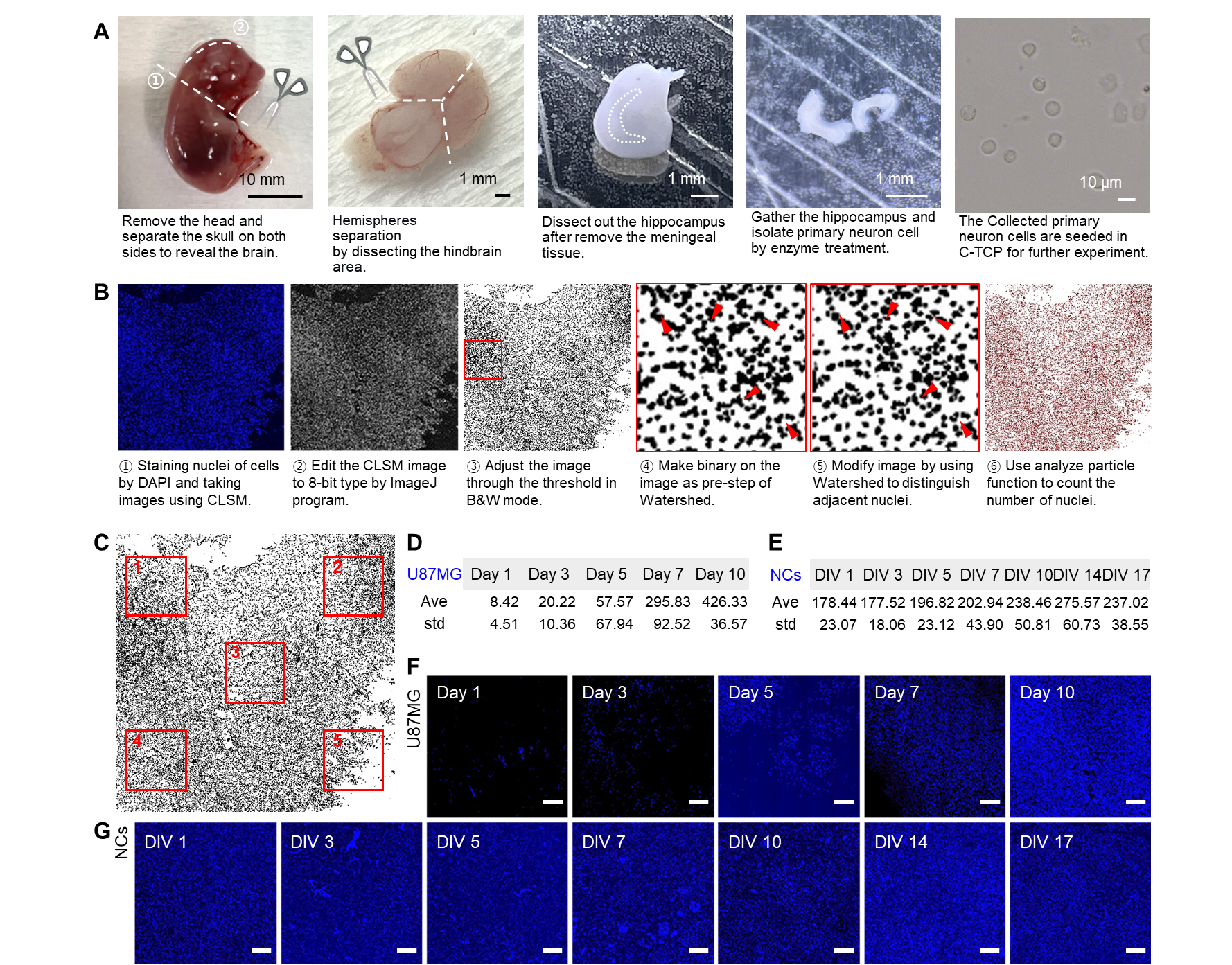


**Supplementary Figure S2: Detailed view of analysis for cell numbers per area.** (A) Procedures of the isolation of hippocampal neuron cells from the brain of rat pups. (B) Process of counting the number of nuclei of cells from CLSM image with DAPI staining using by ImageJ program. Arrowheads indicate representative spots corresponding to dividing proximate cells into single cells. (C) Five areas of counting the number of cell nuclei in CLSM image. The number of U87MG (D) and NCs (E) in 1 mm^2^ during culture dates. CLSM images of U87MG (F) and NCs (G) using for counting the number of cells cultured on C-TCP. Scale bars: 500 µm.


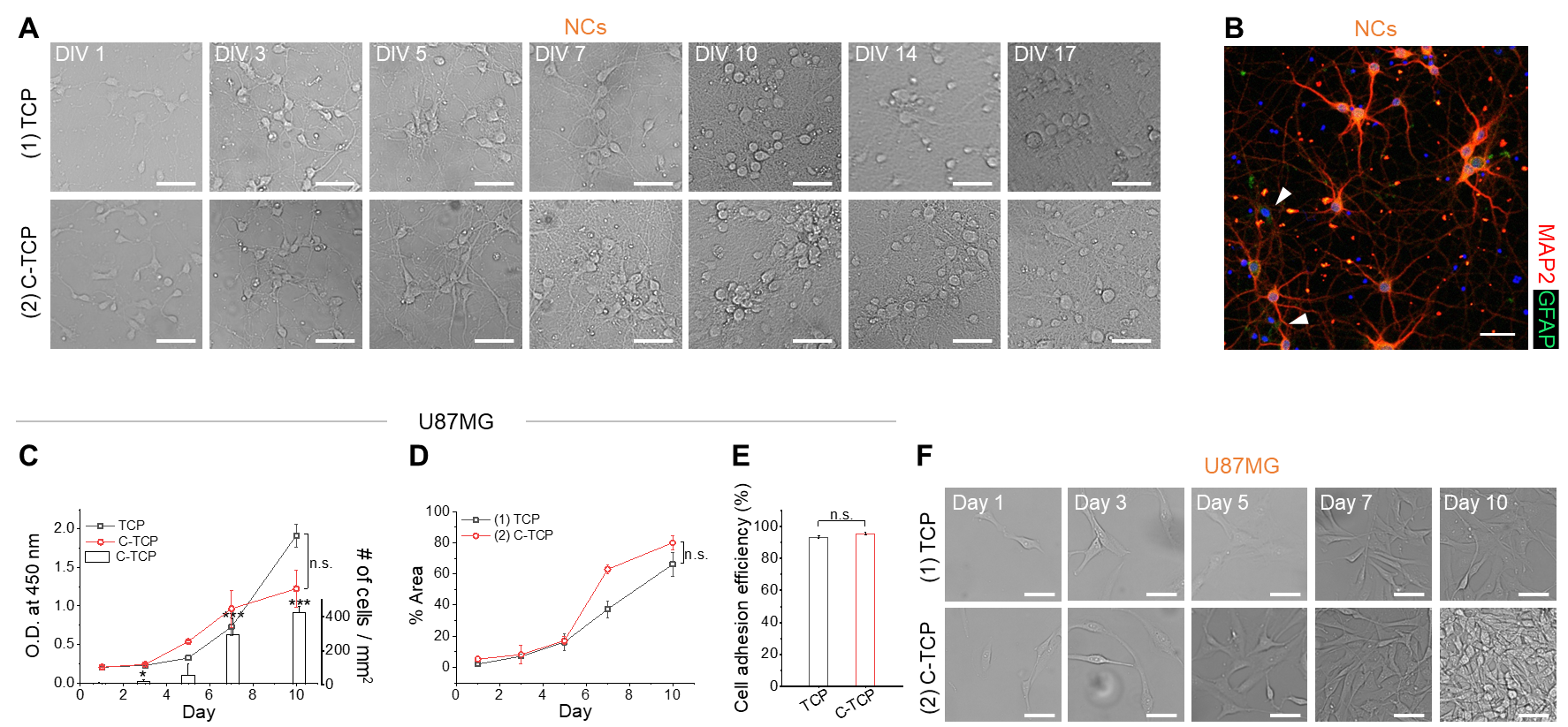
**Supplementary Figure S3: Cytotoxicity analysis of C-TCP using hippocampal neurons and glioblastoma.** (A) Microscopic images of primary neuron cells (NCs) cultured on different types of substrates: TCP vs. C-TCP. Abbreviation: TCP, tissue culture plate; C-TCP, coverslip attached tissue culture plate. (B) A representative CLSM image of DIV 10 NCs immunostained for MAP2 (red) and GFAP (green). Arrowheads indicate GFAP-stained astrocytes. (C) Quantitative results of CCK-8 and number of cells in glioblastoma cells (U87MG). Data were presented as mean ± standard deviation (SD). One-way ANOVA with Tukey’s post hoc test for comparisons. *p < 0.05, **p < 0.01, and ***p < 0.001 indicate statistical significance compared with day 1. n.s., not significant (p > 0.05). (D) Percentage of the cell coverage area determined from microscopic images. Data were presented as mean ± standard deviation (SD). n.s. = not significant (p > 0.05) by one-way ANOVA with Tukey’s post hoc test for comparisons. (E) Cell adhesion efficiency at 24 h after seeding. Data were presented as mean ± standard deviation (SD). n.s. = not significant (p > 0.05) by one-way ANOVA with Tukey’s post hoc test for comparisons. (F) Microscopic images of cell morphology and proliferation during culture dates. Scale bars: 50 µm.


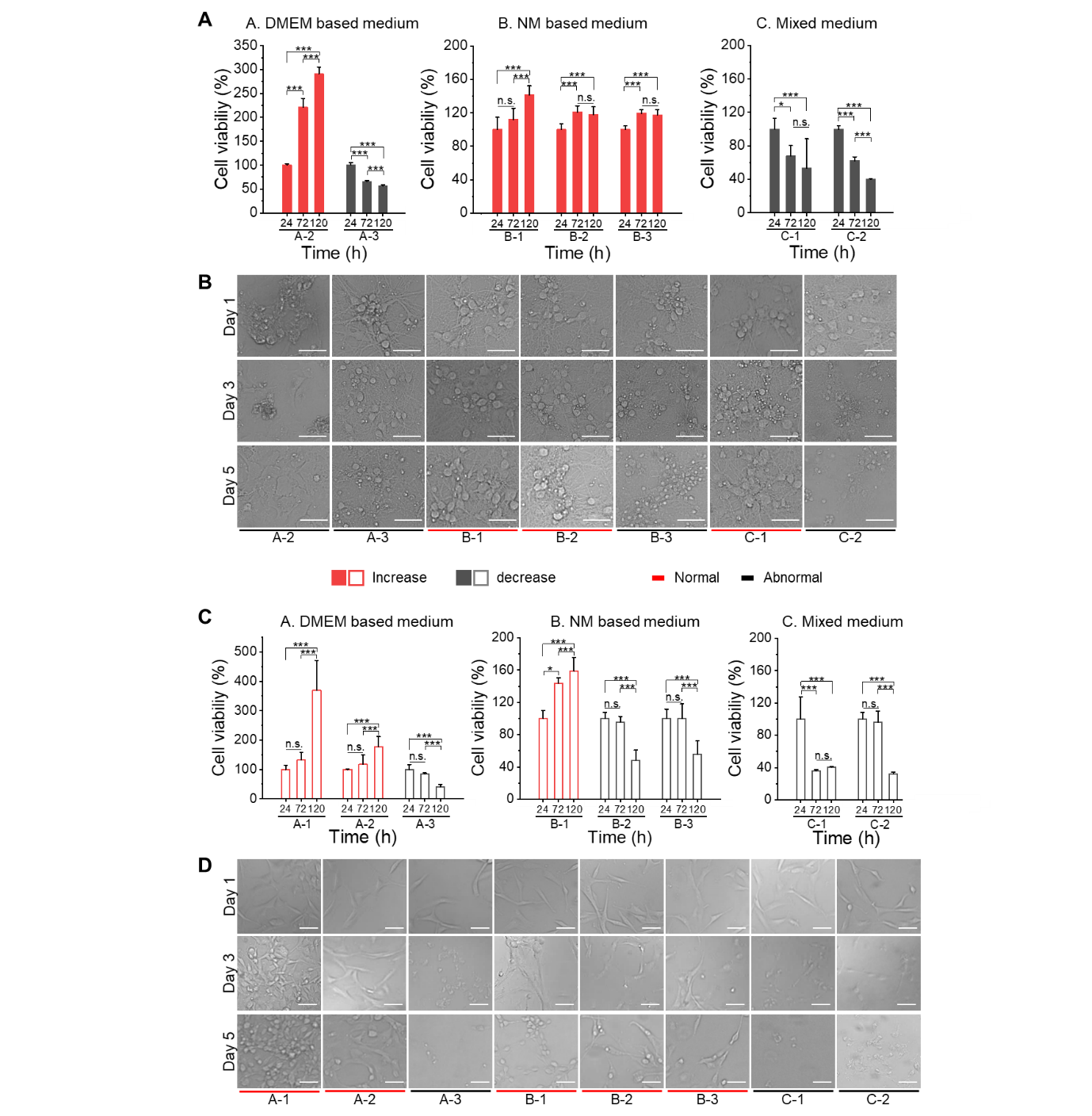


**Supplementary Figure S4: Viability of the two types of cells exposed to the medium with different constituents.** The NCs (A and B) and U87MG (C and D) exhibited differential responses to the medium during culture dates. The abbreviation of each medium is explained in Table S1. (A and C) The effects according to medium composition on cell viability were calculated by CCK-8 assays on days 1, 3, and 5 after changing to the prepared medium. Red-colored and gray-colored bar means increment and decreased tendency, respectively. (B and D) Comparison of prepared medium effect on cell morphologies was observed by optical microscopic images on days 1, 3, and 5 after changing to the prepared medium. Scale bars: 50 µm. The bars under images mean morphological properties of normal (red) and abnormal (black), respectively. All data were presented as mean ± standard deviation (SD). n.s. = not significant (p > 0.05) by one-way ANOVA with Tukey’s post hoc test for multiple comparisons. *p < 0.05, **p < 0.01, and ***p < 0.001. Neuron viability increased in medium A-2 and all NM-based media (group B). Morphological analysis showed neurite and dendrite loss in neurons cultured in A-2, whereas neuronal morphology was largely preserved in group B, with the exception of medium B-3, which induced significant soma shrinkage and cellular protrusions. U87MG cells maintained normal morphology across group B, despite a decrease in viability, particularly in medium B-2.


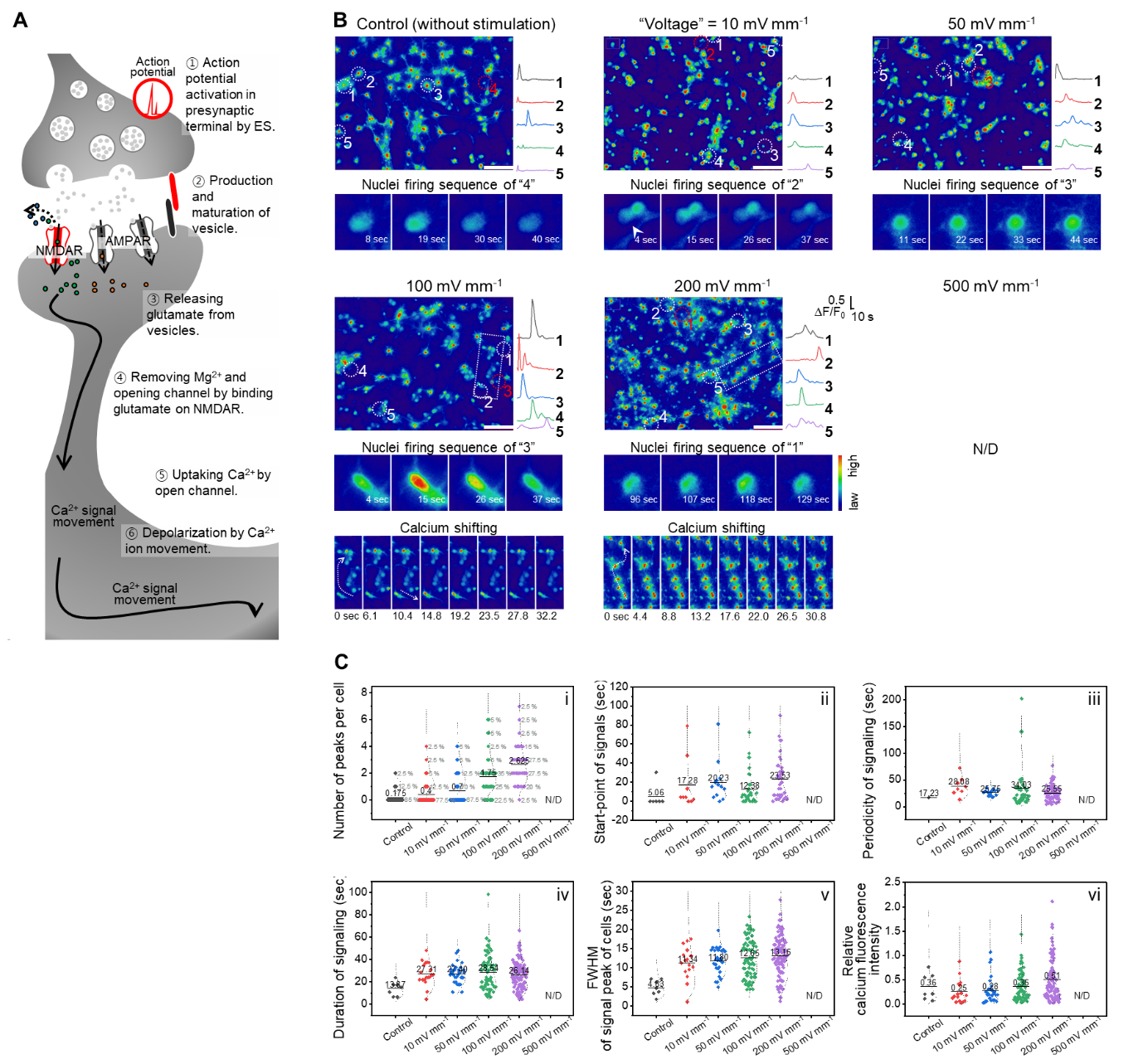


**Supplementary Figure S5: Calcium imaging analysis of voltage-evoked neuronal firing across a range of 10 to 500 mV mm^-1^.** (A) Illustration of calcium ion transfer in neuronal cells induced by electrical stimulation. (B) Representative pseudo-colored calcium images of electrical stimulation response neuronal firing (top row, left). Scale bars: 100 µm. The change in fluorescence response of NCs to different electrical stimulation at five points within the ROI which is depicted by the dotted circle in the representative calcium image is shown in the graph as a ratio (∆F/F_0_) (top row, right). Selected seriate firing neuron images correspond to the red number with red dotted circle inserts in the representative calcium image (middle). A white arrowhead indicates cells in change. Calcium ion tracking images at a position indicated by a dotted rectangle in the top image in a continuous manner (bottom). The dotted arrow is the direction of calcium ion shifting. Relative fluorescence intensity was constructed using a lookup table beside the image. Abbreviation: N/D, not detectable. (C) Calcium signal spectra analysis under different voltage of electrical stimulation: the number of firing peaks per cell (i), starting time point of the signal (ii), periodicity of cell signaling (iii), duration of cell signaling (iv), the full width at half-maximum of firing peak of cell (v), and relative calcium fluorescence intensity (vi). Black bar is the mean value. All electrical stimulation was conducted under 10 Hz for 5 min. Abbreviations: Control, without stimulation. At and above 100 mV mm^-1^, spontaneous neuronal activity was evident through Ca^2+^ translocation across synapses between cells and firing peak numbers per cell. However, at voltages over 500 mV mm^-1^, neurons exhibited irregular morphologies and a lack of Ca^2+^ signaling, implying significant cellular damage.


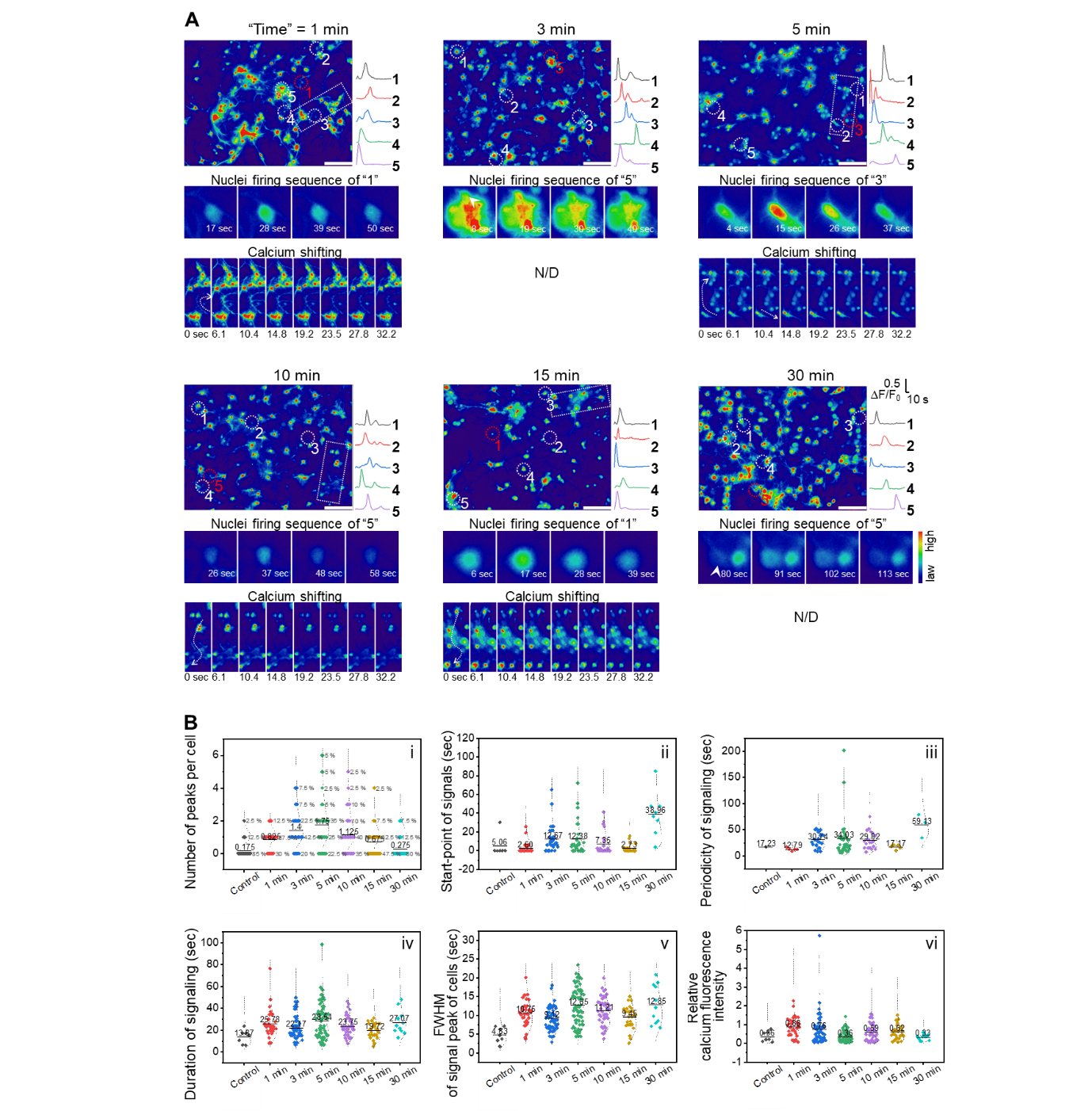


**Supplementary Figure S6: Calcium imaging analysis of activated neuronal networks by time modulation.** (A) Representative pseudo-colored calcium images of electrical stimulation response neuron firing (top row, left). Scale bars: 100 µm. The change in fluorescence response of NCs to different electrical stimulation at five points within the ROI which is depicted by the dotted circle in the representative calcium image is shown in the graph as a ratio (∆F/F_0_) (top row, right). Selected seriate firing neuron images correspond to the red number with red dotted circle inserts in the representative calcium image (middle). A white arrowhead indicates cells in change. Calcium ion tracking images at a position indicated by a dotted rectangle in the top image in a continuous manner (bottom). The dotted arrow is the direction of calcium ion shifting. Relative fluorescence intensity was constructed using a lookup table beside the image. Abbreviation: N/D, not detectable. (B) Calcium signal spectra analysis under the different times of electrical stimulation: the number of firing peaks per cell (i), starting time point of the signal (ii), periodicity of cell signaling (iii), duration of cell signaling (iv), the full width at half-maximum of firing peak of cell (v), and relative calcium fluorescence intensity (vi). Black bar is the mean value. All electrical stimulation was conducted under 100 mV/mm and 10 Hz. Abbreviations: Control, without stimulation. Ca^2+^ shifting through the synapse within the neuronal network was observed intermittently at 1-3 min intervals and then more frequently at 5-15 min, but not observed beyond 30 min post-stimulation. Quantitative analysis indicates that stimulation periods exceeding 3 min resulted in one or more firing peaks, while durations over 10 min led to a decline in activity.


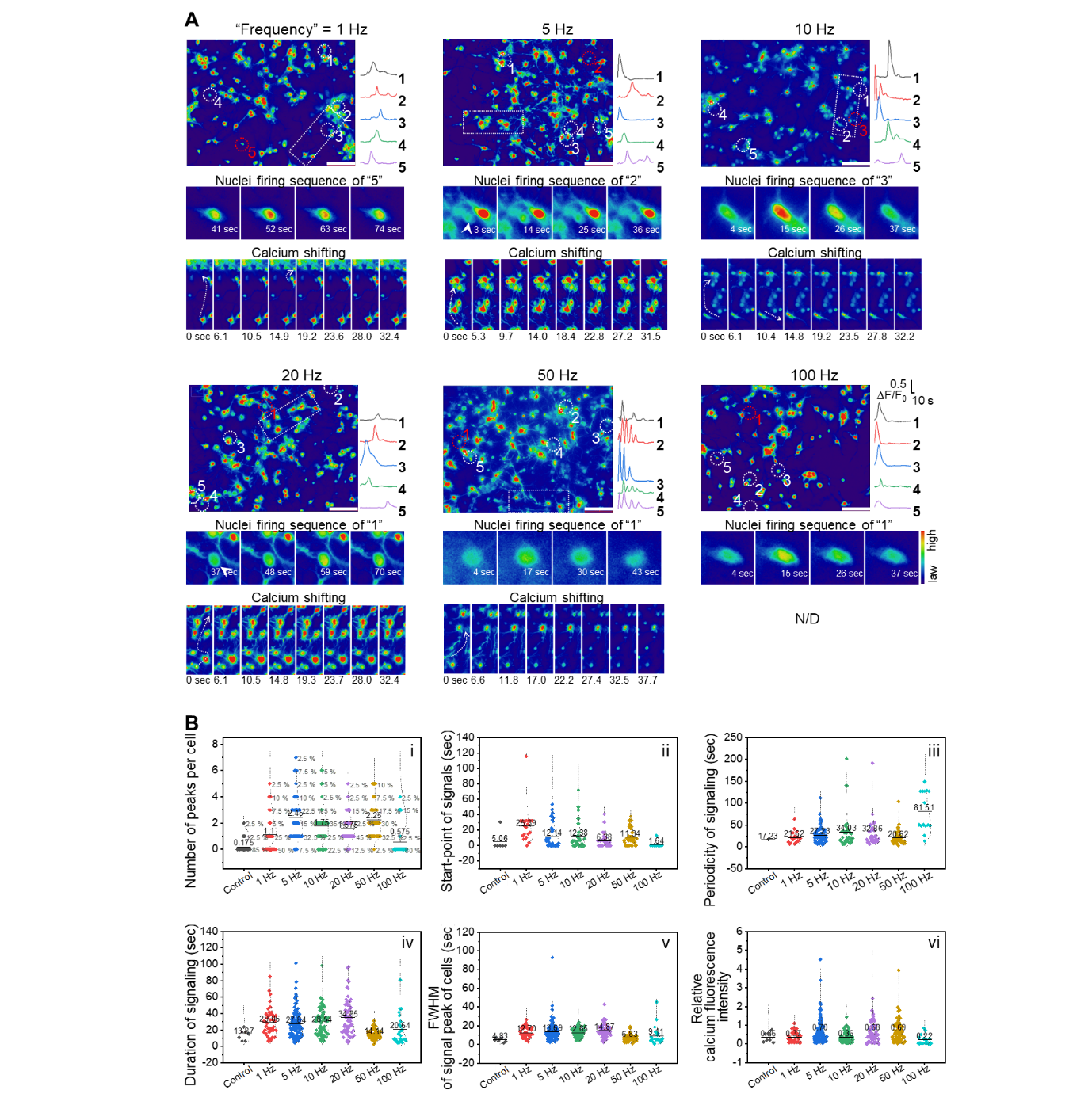


**Supplementary Figure S7: Calcium imaging analysis of activated neuronal networks by different frequencies of electrical stimulation.** (A) Representative pseudo-colored calcium images of electrical stimulation response neuron firing (top row, left). Scale bars: 100 µm. The change in fluorescence response of NCs to different electrical stimulation at five points within the ROI which is depicted by the dotted circle in the representative calcium image is shown in the graph as a ratio (∆F/F_0_) (top row, right). Selected seriate firing neuron images correspond to the red number with red dotted circle inserts in the representative calcium image (middle). A white arrowhead indicates cells in change. Calcium ion tracking images at a position indicated by a dotted rectangle in the top image in a continuous manner (bottom). The dotted arrow is the direction of calcium ion shifting. Relative fluorescence intensity was constructed using a lookup table beside the image. Abbreviation: N/D, not detectable. (B) Calcium signal spectra analysis under the different frequencies of electrical stimulation: the number of firing peaks per cell (i), starting time point of the signal (ii), periodicity of cell signaling (iii), duration of cell signaling (iv), the full width at half-maximum of firing peak of cell (v), and relative calcium fluorescence intensity (vi). Black bar is the mean value. All electrical stimulation was conducted under 100 mV/mm for 5 min. Abbreviations: Control, without stimulation.


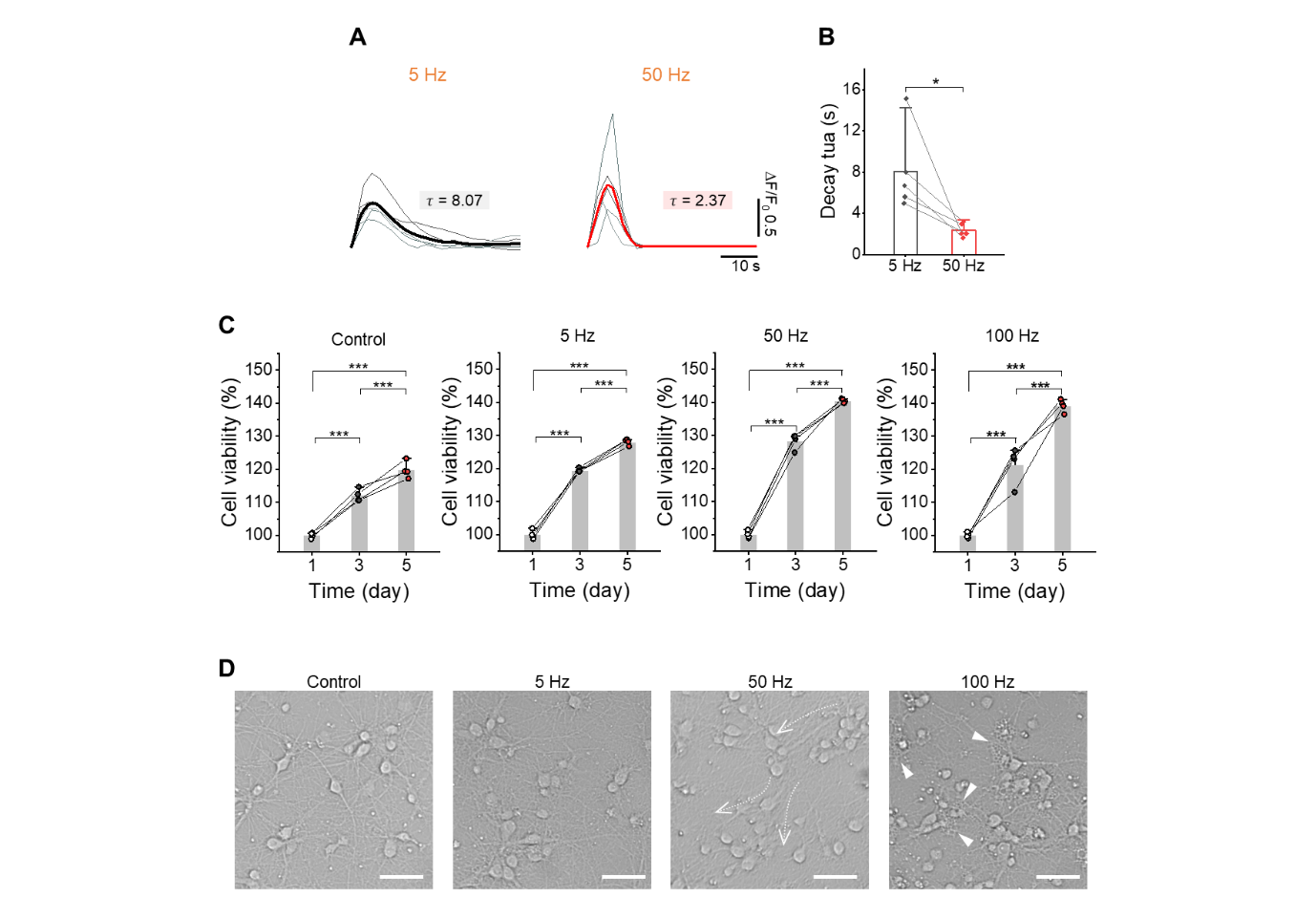


**Supplementary Figure S8: Relative neuronal metabolic activities after electrical stimulation in different frequencies.** (A) Representative traces of the calcium ion triggered by electrical stimulation (5 Hz; normal, 50 Hz; abnormal). Thick lines represent the average of the responses. (B) Graph showing the calcium decay constant (decay tau, τ), calculated from calcium signal responses following electrical stimulation. Data represent mean ± standard deviation (SD). Statistical significance was assessed using one-way ANOVA with Tukey’s post hoc test; *p < 0.05. (C) Relative cell metabolic activity under different electrical stimulation conditions. Data were presented as mean ± standard deviation (SD). One-way ANOVA with Tukey’s post hoc test for comparisons. ***p < 0.001. (D) Neuronal network morphology on 1 day after electrical stimulation. The obvious direction of NCs after 50 Hz electrical stimulation is visible and marked by dotted arrows. Clear dendrite damage is shown in NCs after 100 Hz electrical stimulation and is indicated by the arrowheads. Scale bars: 50 µm. Abbreviations: Control, without stimulation.


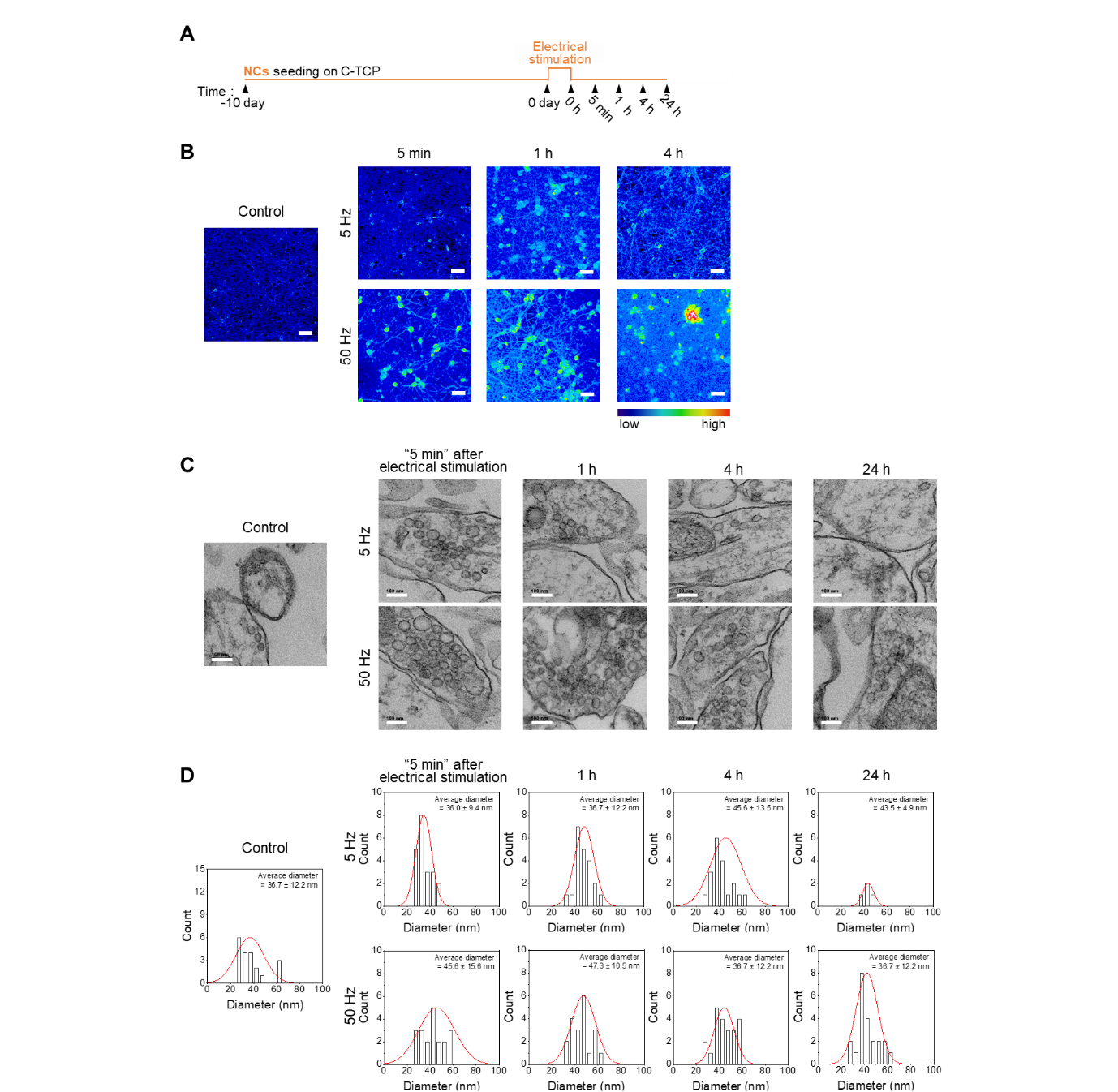


**Supplementary Figure S9: Glutamate and vesicle production events in neuronal cells after the electrical stimulation.** (A) Experimental strategy of intracellular glutamate and vesicle production analysis. (B) Fluorescence microscopic images of glutamate from NCs after electrical stimulation in different manners. The color bar indicates the scale of relative fluorescence intensity. Scale bars: 50 µm. (C) Representative TEM images of clear vesicles from cultured NCs after electrical stimulation. Scale bars: 100 nm. (D) Diameter distributions of vesicles from NCs at different time intervals after electrical stimulation. Averaged vesicle diameters are described in the graph, respectively. ANOVA tests showed no significant size changes up to 24 h at 50 Hz stimulation, suggesting vesicle maturation in preparation for exocytosis. Abbreviations: Control, without stimulation.


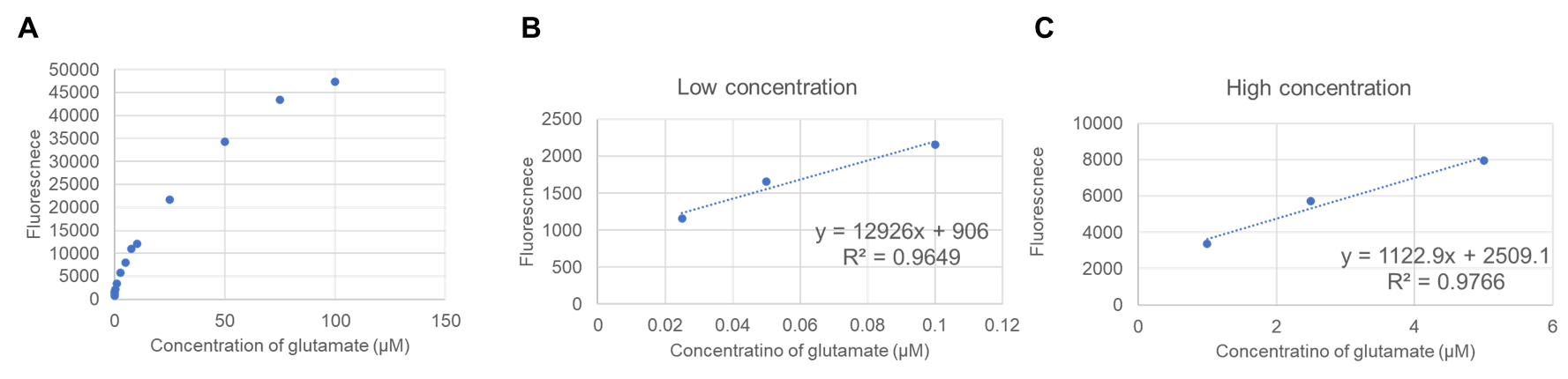


**Supplementary Figure S10: Standard curves for glutamate quantification using the Amplex^TM^ Red assay.** (A) Full-range calibration curve generated using glutamate standards from 0 to 100 µM. (B) Expanded view of the low-concentration range with linear regression (R² = 0.9649), used to determine sensitivity at minimal glutamate levels. (C) Expanded view of the high-concentration range with corresponding linear regression (R² = 0.9766), applied for samples with elevated glutamate concentrations.


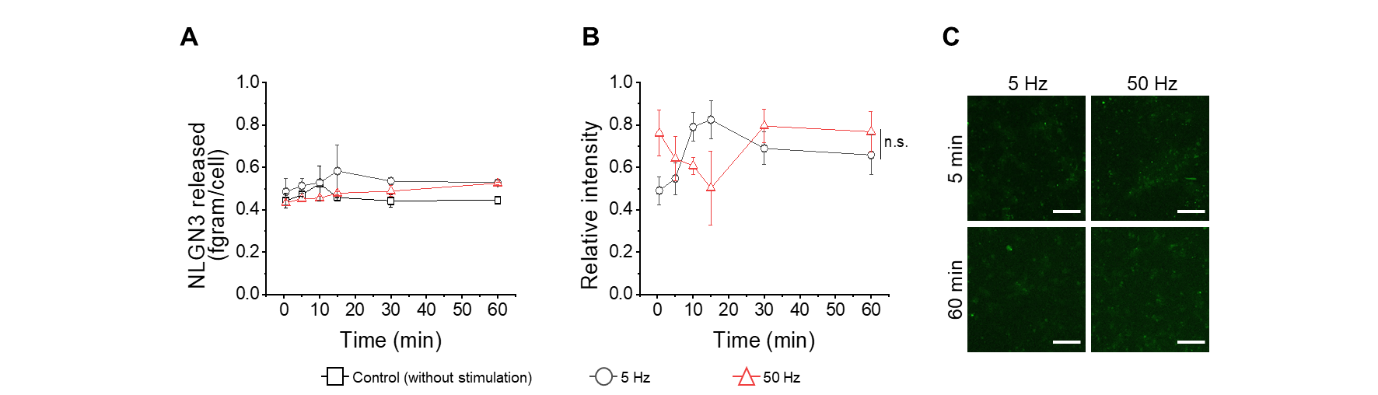


**Supplementary Figure S11: Analysis of neurotrophic factor release from electrically stimulated neuronal networks.** (A) Detection of NLGN3 release from neuronal network after electrical stimulation in a time-dependent manner. Quantitative immunofluorescence intensity analysis (B) and representative images (C) of the neuronal network after electrical stimulation. Scale bars: 100 µm. Abbreviations: Control, without stimulation. All data were presented as mean ± standard deviation (SD). Abbreviation: n.s., not significant (p > 0.05).


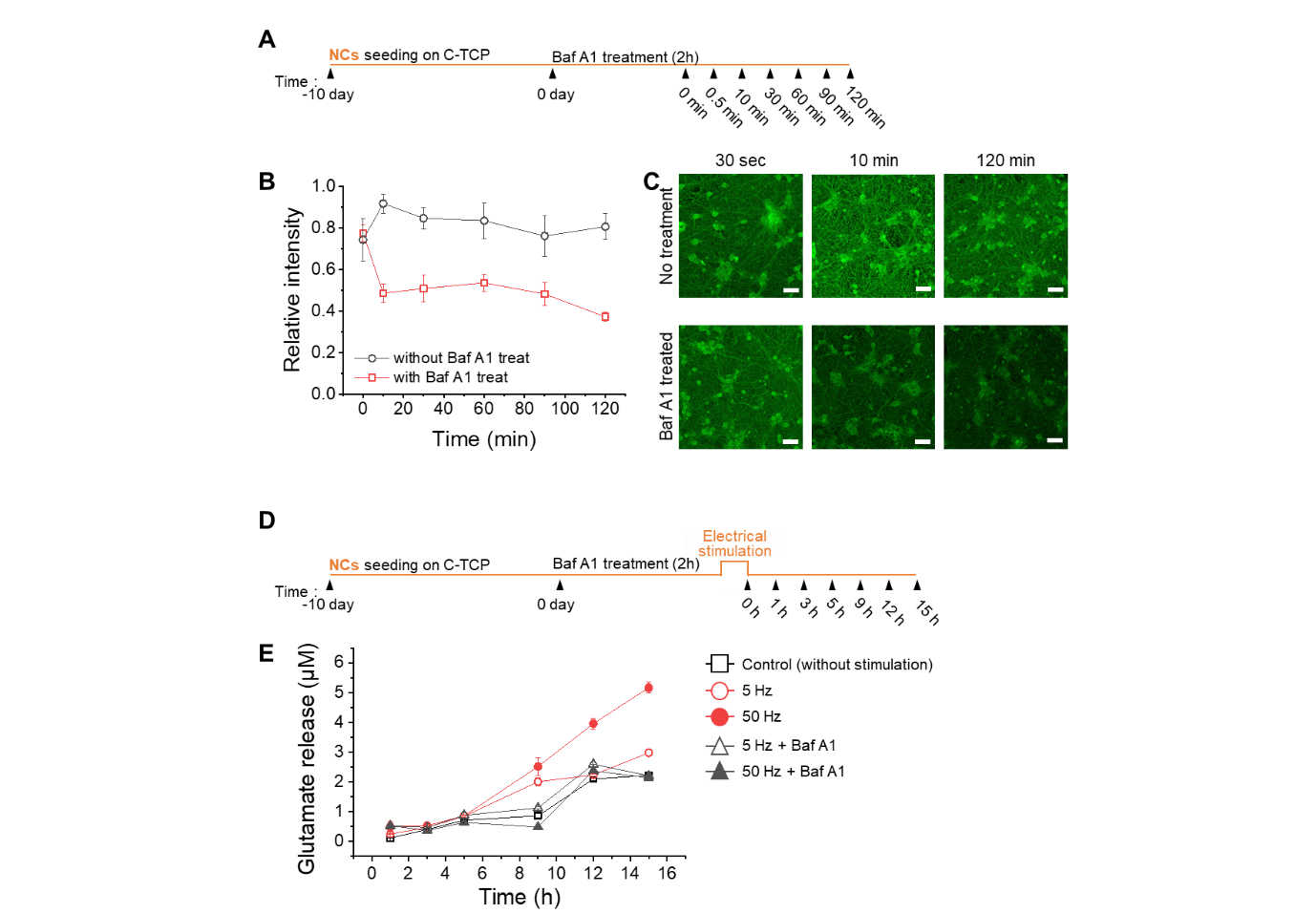


**Supplementary Figure S12: Exocytic suppressed neuron cells after Baf A1 treatment as glutamate inhibitor.** (A) Summarized process of an experiment for Baf A1 effect analysis. (B) Analysis of immunofluorescence intensity after Baf A1 treatment for time-dependent glutamate depletion effectivity in NCs. (C) Representative glutamate immunofluorescence images with Baf A1 treatment in time series. Time-course studies of glutamate release under Baf A1 treatment revealed an initial rapid decrease at dendritic regions, with a gradual reduction at the soma attributed to the combined effects of Baf A1 and the glutamate-free medium. By 2 h, glutamate levels were sufficiently negligible with Baf A1 treatment. Scale bars: 50 µm. (D) Summarized process of study for glutamate release from the neuronal network after electrical stimulation. (E) Glutamate release from the neuronal network after electrical stimulation under the glutamate-deplete medium. The results indicated sustained glutamate release at 50 Hz and a modest increase at 5 Hz compared to unstimulated and Baf A1-treated control. These findings validate the use of Baf A1 as an effective glutamate-release inhibitor. Abbreviations: Control, without stimulation and + Baf A1, with Baf A1 treatment. All data were presented as mean ± standard deviation (SD).


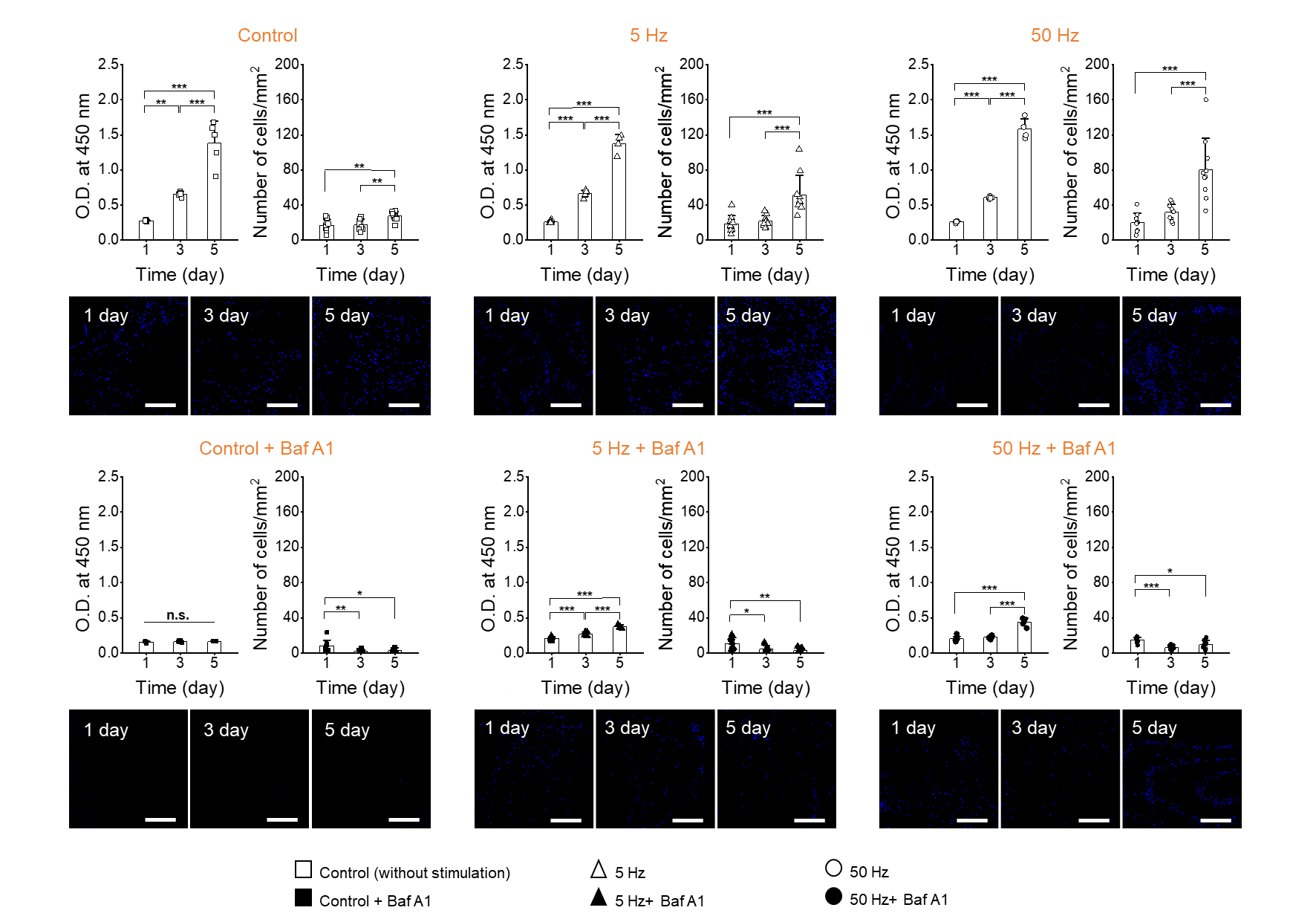


**Supplementary Figure S13: The progression of glioblastoma co-cultured with a hyperexcited neuronal network under the glutamate-deplete environment.** CCK-8 assay (top row, left), number of cells (top row, right), and CLSM images (bottom row) of glioblastoma co-cultured under the glutamate-deplete environment with the electrical stimulated neuronal network. Scale bars: 1 mm. Abbreviations: Control, without stimulation and + Baf A1, with Baf A1 treatment. All data were presented as mean ± standard deviation (SD). One-way ANOVA with Tukey’s post hoc test was used for multiple comparisons. *p < 0.05, **p < 0.01, and ***p < 0.001.


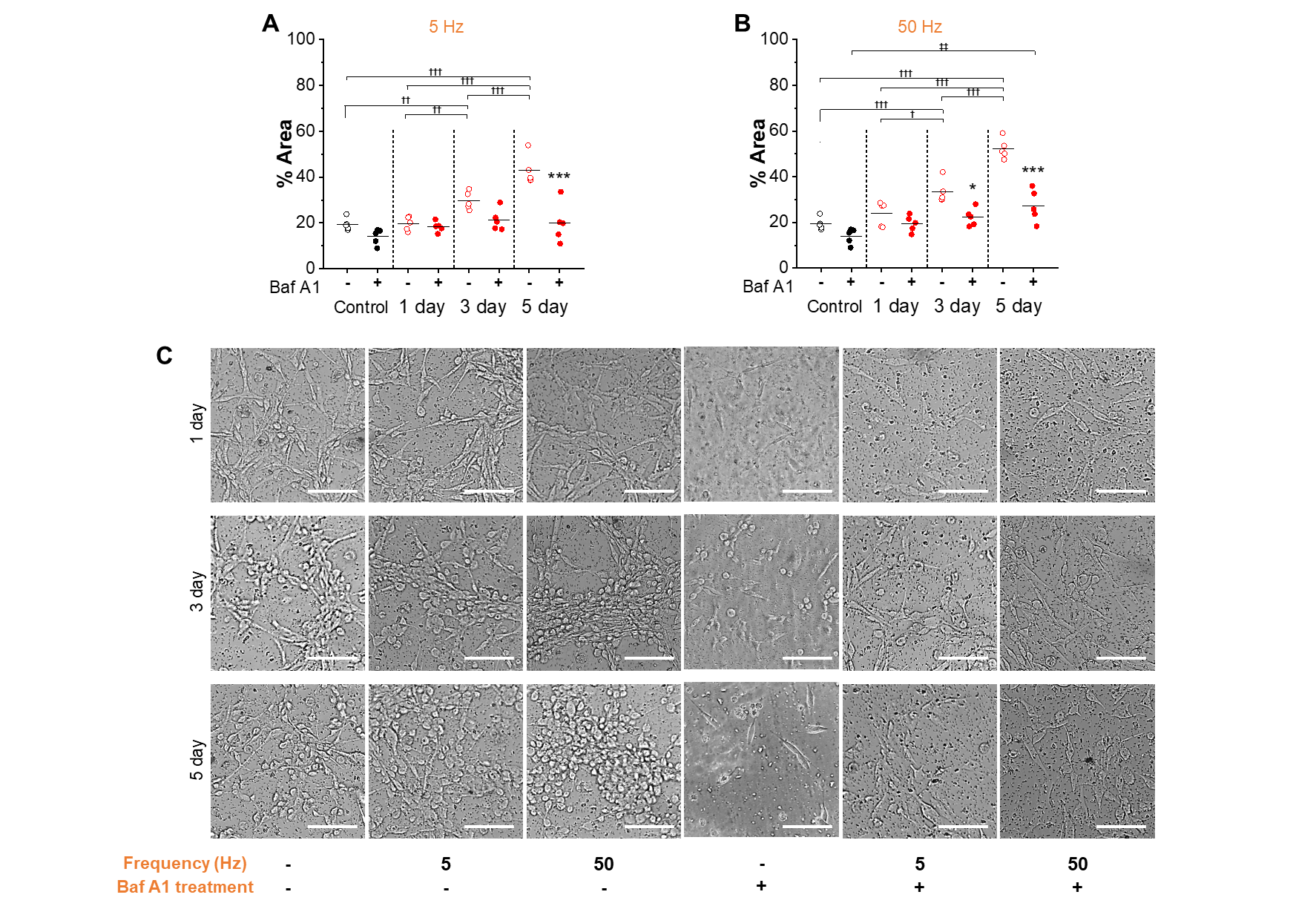


**Supplementary Figure S14: Glioblastoma progression co-cultured with a hyperexcited neuronal network under the glutamate-deplete environment.** Average U87MG area coverage analyses after co-culture with electrically stimulated neuronal network in 5 (A) and 50 Hz (B). (C) Optical microscopic images of U87MG co-cultured with stimulated neuronal network in the glutamate-deplete medium. Scale bars: 100 µm. Abbreviations: Control, without stimulation and + Baf A1, with Baf A1 treatment. All data were presented as mean ± standard deviation (SD). One-way ANOVA with Tukey’s post hoc test was used for multiple comparisons. *p < 0.05, **p < 0.01, and ***p < 0.001 vs. without Baf A1 treatment of each group; †p < 0.05, ††p < 0.01, and †††p < 0.001 indicate statistical significance compared within without Baf A1 treatment group; ‡p < 0.05, ‡‡p < 0.01, and ‡‡‡p < 0.001 indicate statistical significance compared within with Baf A1 treatment group.


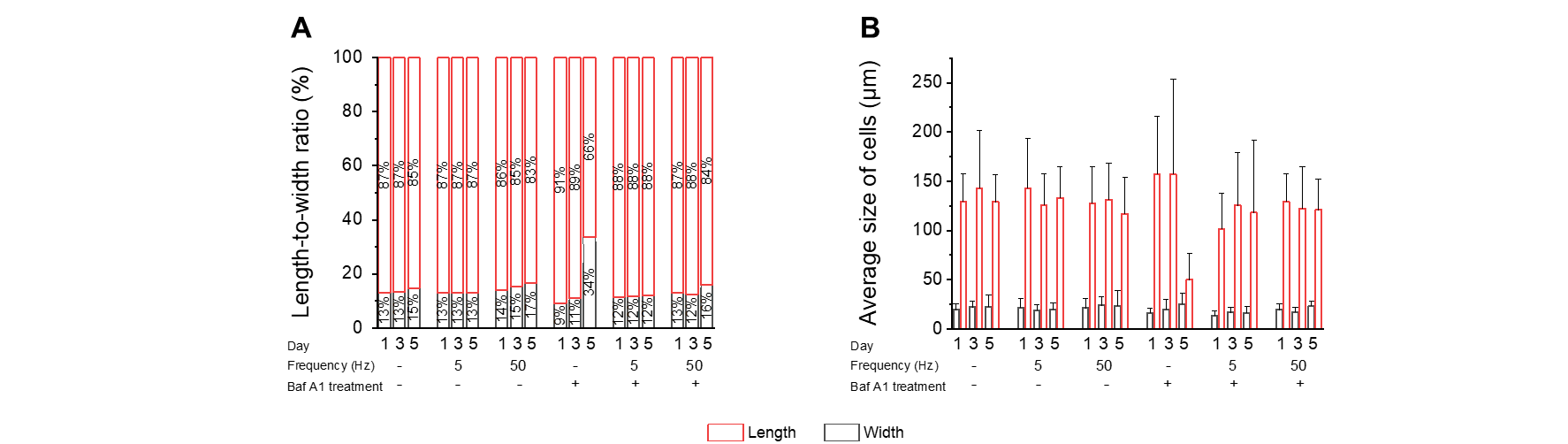


**Supplementary Figure S15: Change of glioblastoma phenotype co-cultured with a hyperexcited neuronal network under the glutamate-deplete environment.** Stacked column of length-to-width ratio (A) and bar graph for average length and width (B) of glioblastoma cells during 5 cultivation days.


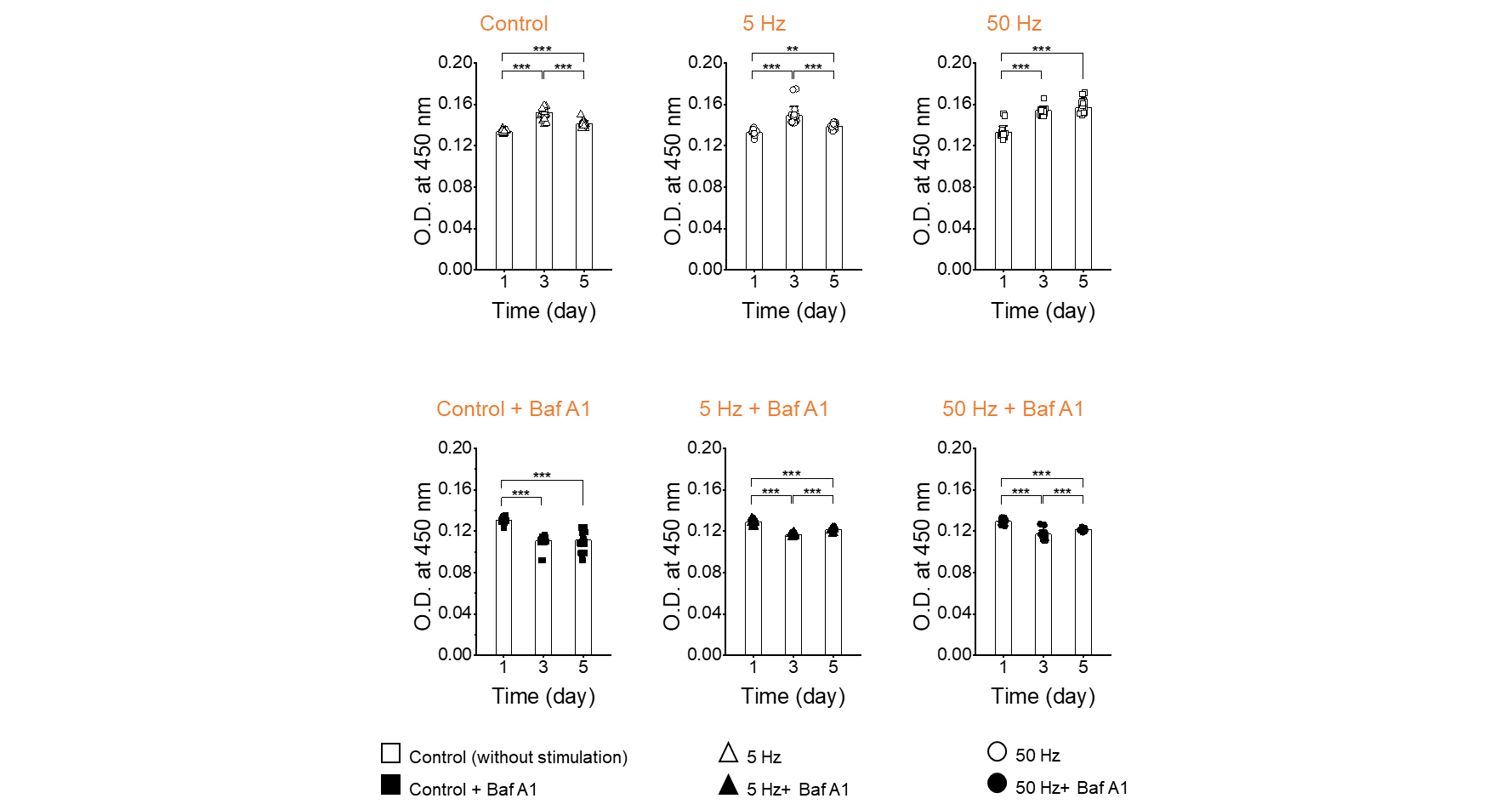


**Supplementary Figure S16: The progression of GL261 co-cultured with a hyperexcited neuronal network.** CCK-assay of GL261 co-cultured under the glutamate-deplete environment with the electrical stimulated neuronal network. All data were presented as mean ± standard deviation (SD). One-way ANOVA with Tukey ’s post hoc test was used for multiple comparisons. *p < 0.05, **p < 0.01, and ***p < 0.001.


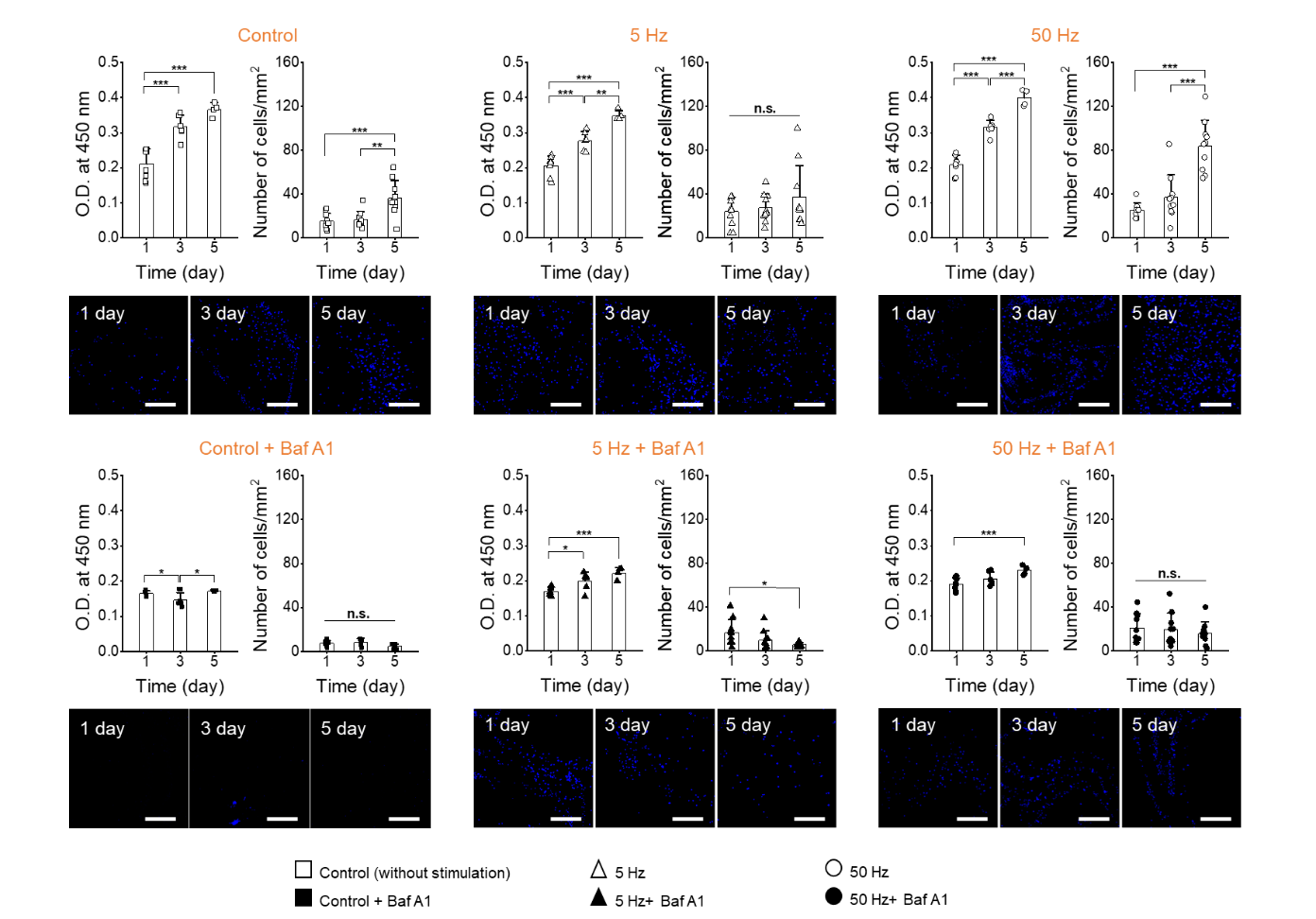


**Supplementary Figure S17: The recurrence of glioblastoma co-cultured with a hyperexcited neuronal network after chemotherapy.** CCK-8 assay (top row, left), number of cells (top row, right), and CLSM images (bottom row) of glioblastoma after chemotherapy co-cultured with electrical stimulated neuronal network. Scale bars: 1 mm. Abbreviations: Control, without stimulation and + Baf A1, with Baf A1 treatment. All data were presented as mean ± standard deviation (SD). n.s. = not significant (p > 0.05) by one-way ANOVA with Tukey’s post hoc test for multiple comparisons. *p < 0.05, **p < 0.01, and ***p < 0.001.


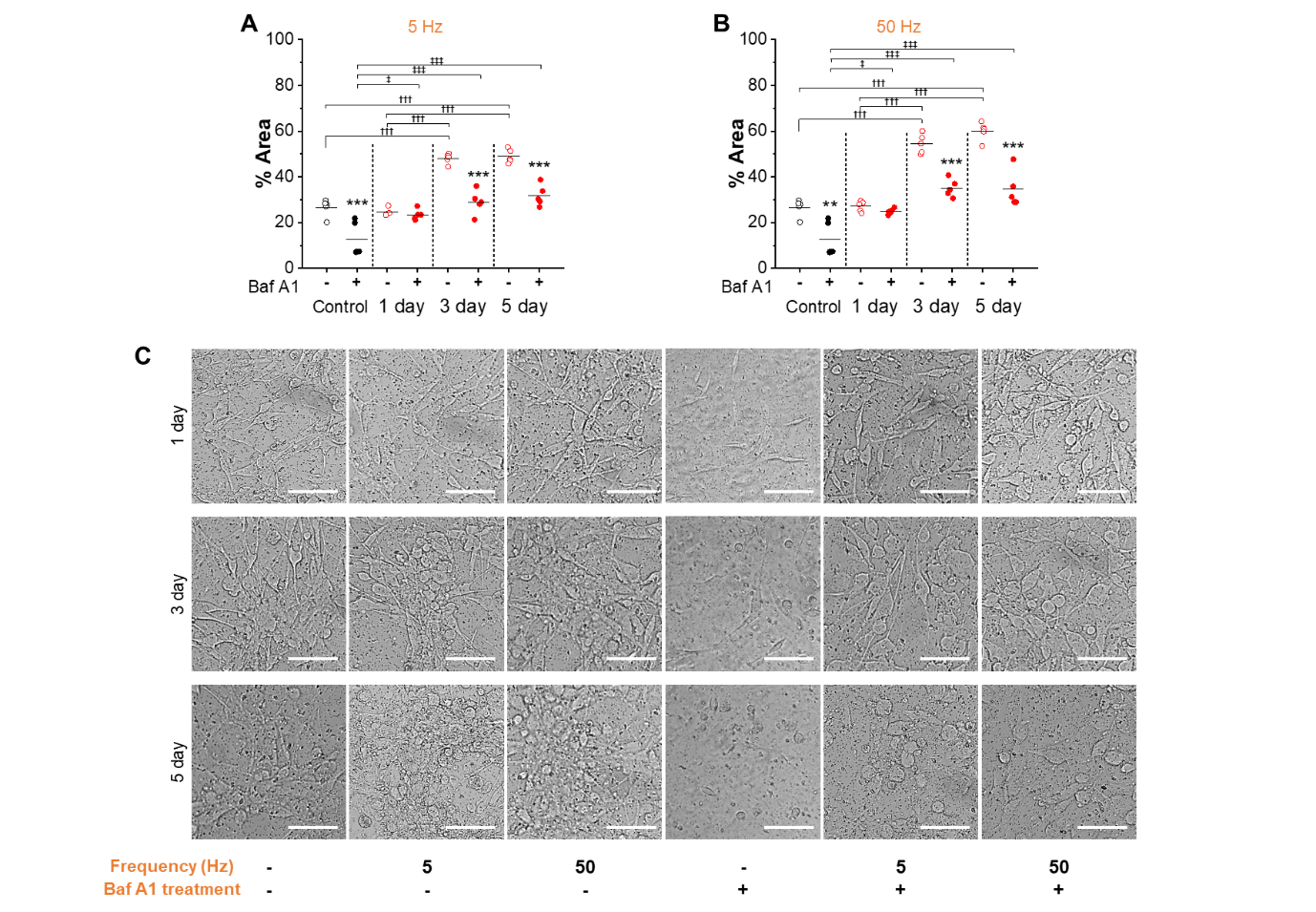


**Supplementary Figure S18: The glioblastoma recurrence co-cultured with a hyperexcited neuronal network after chemotherapy.** Average U87MG area coverage analyses after co-culture with electrically stimulated neuronal network in 5 (A) and 50 Hz (B). (C) Optical microscopic images of TMZ-treated U87MG co-cultured with stimulated neuronal network. Scale bars: 100 µm. Abbreviations: Control, without stimulation and + Baf A1, with Baf A1 treatment. All data were presented as mean ± standard deviation (SD). One-way ANOVA with Tukey’s post hoc test was used for multiple comparisons. *p < 0.05, **p < 0.01, and ***p < 0.001 vs. without Baf A1 treatment of each group; †p < 0.05, ††p < 0.01, and †††p < 0.001 indicate statistical significance compared within without Baf A1 treatment group; ‡p < 0.05, ‡‡p < 0.01, and ‡‡‡p < 0.001 indicate statistical significance compared within with Baf A1 treatment group.


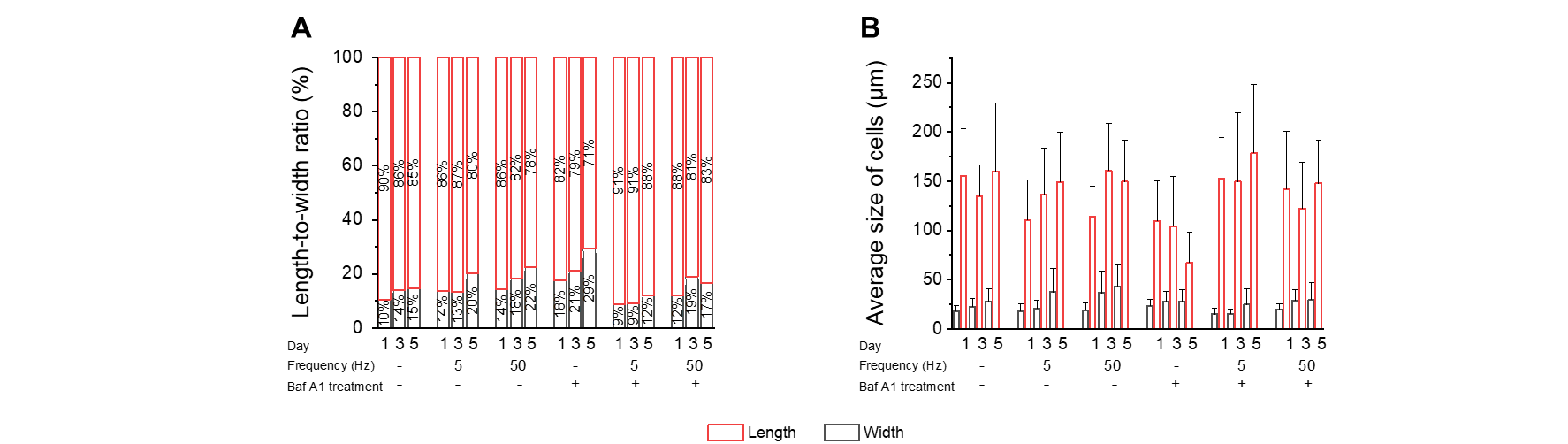


**Supplementary Figure S19: Change of glioblastoma phenotype co-cultured with a hyperexcited neuronal network after chemotherapy.** Stacked column of length-to-width ratio (A) and bar graph for average length and width (B) of glioblastoma cells during 5 cultivation days.

**Supplementary Table 1: Components information of medium**.

| Main category | Subcategory | Components of medium |
| --- | --- | --- |
| (A)DMEM based medium | (A-1)  DMEM+FBS+P/S | - DMEM (Dulbecco's Modified Eagle Medium; 89 %) - FBS (Fetal Bovine Serum; 10 %) - P/S (Penicillin-streptomycin; 1 %) |
|  | (A-2)  L-DMEM+FBS+P/S | - L-DMEM (DMEM without l-glutamate; 89 %) - FBS (Fetal Bovine Serum; 10 %) - P/S (Penicillin-streptomycin; 1 %) |
|  | (A-3)  L-DMEM+P/S | - L-DMEM (DMEM without l-glutamate; 99 %) - P/S (Penicillin-streptomycin; 1 %) |
| (B) NM based medium | (B-1)  NM+Gtmax+B27+P/S | - NM (Neurobasal medium; 97 %) - Gtmax (Glutamax; 1 %) - B27 (B-27™ Supplement; 1 %) - P/S (Penicillin-streptomycin; 1 %) |
|  | (B-2)  NM+B27+P/S | - NM (Neurobasal medium; 98 %) - B27 (B-27™ Supplement; 1 %) - P/S (Penicillin-streptomycin; 1 %) |
|  | (B-3)  NM+P/S | - NM (Neurobasal medium; 99 %) - P/S (Penicillin-streptomycin; 1 %) |
| (C) Mixed medium | (C-1)  L-DMEM+NM+B27+P/S | - L-DMEM (DMEM without l-glutamate; 49 %) - NM (Neurobasal medium; 49 %) - B27 (B-27™ Supplement; 1 %) - P/S (Penicillin-streptomycin; 1 %) |
|  | (C-2)  L-DMEM+NM+P/S | - L-DMEM (DMEM without l-glutamate; 49.5 %) - NM (Neurobasal medium; 49.5 %) - P/S (Penicillin-streptomycin; 1 %) |
